# Synchrotron-based X-ray 3D phase contrast imaging and analysis of transmural myocardial tissue from heart failure patients

**DOI:** 10.1038/s41598-025-04012-5

**Published:** 2025-07-16

**Authors:** Nikola Skreb, Filip Loncaric, Kan Yan Chloe Li, Anne Bonnin, Hector Dejea, Patricia Garcia-Canadilla, Ivana Ilic, Hrvoje Gasparovic, Davor Milicic, Bart Bijnens, Andrew C. Cook, Ivo Planinc, Maja Cikes

**Affiliations:** 1 Department of Cardiovascular Diseases, University of Zagreb School of Medicine, University Hospital Centre Zagreb, Kispaticeva 12, 10000 Zagreb, Croatia; 2 Institute of Cardiovascular Science, University College London, London, UK; 3 Swiss Light Source, Paul Scherrer Institute, Villigen, Switzerland; 4 European Synchrotron Radiation Facility, Grenoble, France; 5 Cardiovascular Diseases and Child Development, Sant Joan de Déu Research Institute (IRSJD), Esplugues de Llobregat, Spain; 6 Barcelona Centre for Maternal-Fetal and Neonatal Medicine (BCNatal), Hospital Sant Joan de Déu and Hospital Clínic, University of Barcelona, Barcelona, Spain; 7 Department of Pathology and Cytology, University of Zagreb School of Medicine, University Hospital Centre Zagreb, Zagreb, Croatia; 8 Department of Cardiac Surgery, University of Zagreb School of Medicine, University Hospital Centre Zagreb, Zagreb, Croatia; 9 Catalan Institution for Research and Advanced Studies, ICREA, Barcelona, Spain; 10 Universitat Pompeu Fabra, Barcelona, Spain

**Keywords:** Heart failure, Cardiomyopathy, Heart transplantation, Left ventricular assist device, X-ray phase contrast imaging, Histopathology, Medical imaging, Cardiology

## Abstract

Synchrotron-based X-ray phase contrast imaging (X-PCI) is a non-destructive imaging modality that can provide high resolution three-dimensional (3D) visualisation of transmural myocardial tissue, collagen matrix reconstruction, and quantification of myocyte aggregate orientation (‘myomapping’). We aimed to use X-PCI to analyse microstructural features in transmural myocardial samples from patients with advanced heart failure. Six patients were included: two receiving a left ventricular assist device (LVAD) for ischaemic (ICM) and dilated cardiomyopathy (DCM), and four undergoing heart transplantation (HTx), two for the ICM, one for DCM and one for toxic cardiomyopathy. Samples were obtained by left ventricular (LV) apical coring (LVAD group) or from the LV free wall of the explanted hearts (HTx group) and imaged by X-PCI using a multi-scale setup (maximal resolution at 0.65 µm pixel size). The 3D image datasets were analysed via two-dimensional orthogonal cuts in different layers. Visualisation and quantification of the myocyte aggregates orientation showed a disruption in epicardial-to-endocardial transition in DCM, whereas the collagen matrix reconstruction identified characteristic fibrosis patterns amongst different HF aetiologies. In conclusion, X-PCI is a 3D imaging method that can extend the amount of information available from ex-vivo tissue analysis and, as an addition to multimodal imaging protocols, potentially improve disease phenotyping.

**Supplementary Information:**

The online version contains supplementary material available at 10.1038/s41598-025-04012-5.

## Introduction

Cardiomyopathies represent a heterogenous group of diseases of the myocardium that manifest with both structural and functional alterations, usually in the form of ventricular hypertrophy or dilatation^1^. The contemporary approach to managing complex cardiac disease involves integration of heterogeneous data, from clinical presentation to genetic information, laboratory markers, and non-invasive and invasive cardiac imaging. Multimodality cardiac imaging plays a crucial role in identifying structural changes in the myocardium, with different imaging modalities serving as diagnostic tools contributing towards improved understanding of the involved pathophysiology and forming the basis for a tailored approach to patient management.

Synchrotron-based X-ray phase contrast imaging (X-PCI), a recent imaging modality in the biomedical field including cardiovascular research, is a non-destructive imaging modality that, unlike classical histopathology, does not require complex tissue preparation^2–16^. X-PCI has shown potential in providing high resolution (HR) three-dimensional (3D) visualisation of biological tissue on the macro- and microstructural level^14–17^, also enabling full cardiac tissue analysis ex-vivo, from the epicardium to endocardium^2–19^. We aimed to use X-PCI to image, analyse and compare microstructural features and histopathological differences in transmural cardiac tissue samples from various primary and secondary cardiomyopathies and integrate these findings with the standard diagnostic tools utilised in heart failure (HF), to further improve disease phenotyping.

## Results

### Patient characteristics

**Fig. 1:**
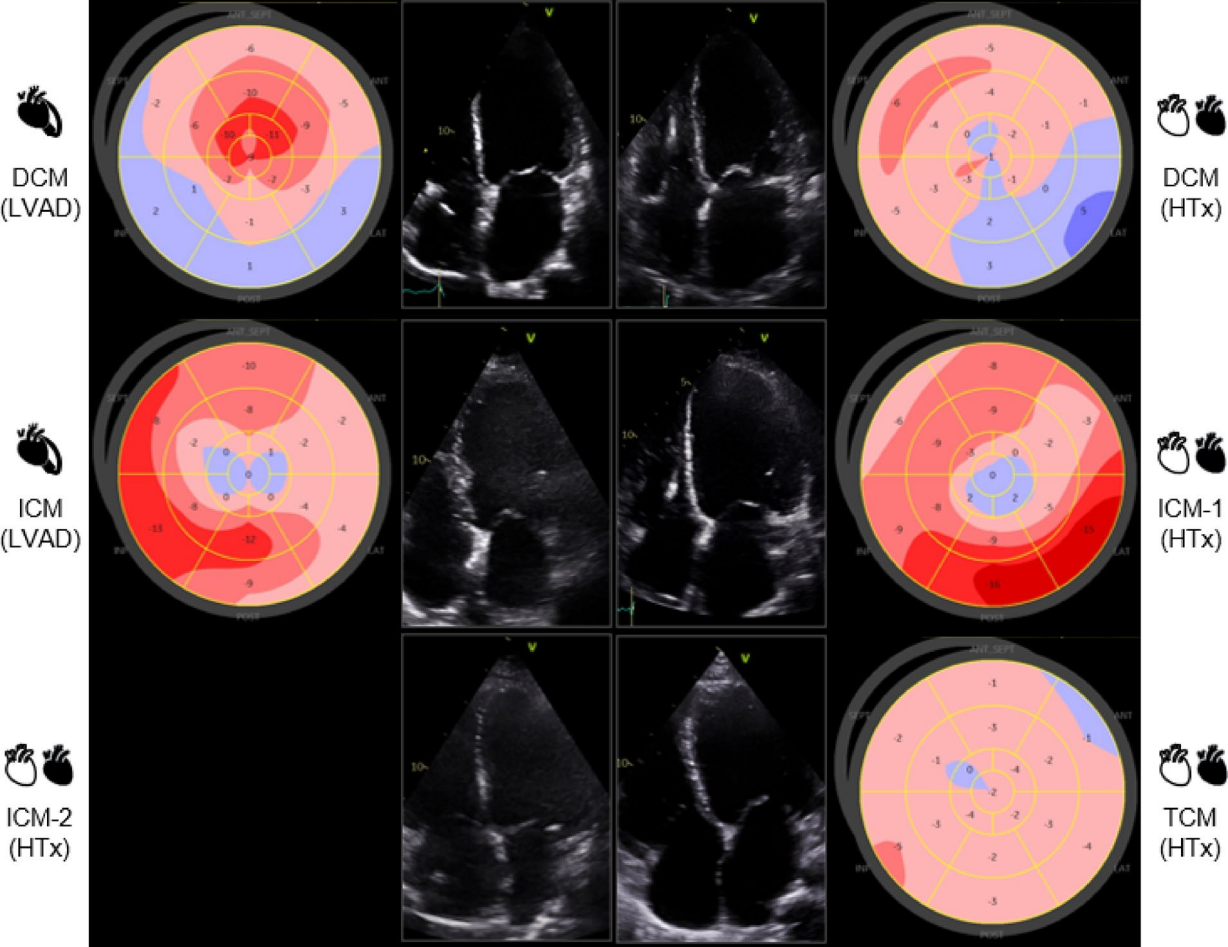
Non-invasive imaging phenotypes. The four-chamber apical view (central images) shows that the structural phenotype of the heart is similar in end-stage HF (i.e., eccentric remodelling, enlarged LV end-diastolic diameter and volume, enlarged LA), whereas the global longitudinal strain bullseye plots (left and right columns) show different functional phenotypes associated with disease aetiologies (i.e., regional differences in peak systolic deformation in ischaemic cardiomyopathy related to the coronary blood flow distribution vs. global deformation impairment in dilated and toxic cardiomyopathies). Patient ICM-2 had a poor echocardiographic window with image quality that did not permit deformation analysis. *HF* heart failure, *LV* left ventricle, *LA* left atrium, *DCM* dilated cardiomyopathy, *ICM* ischaemic cardiomyopathy, *TCM* toxic cardiomyopathy.

General patient data, echocardiography and laboratory characteristics are shown in Table 1. All six patients were male, and their ages ranged from 28 years old (toxic cardiomyopathy (TCM)) to 67 years old (ischaemic cardiomyopathy (ICM)) at the time of treatment. Comorbidities (i.e., hyperlipidaemia, diabetes, arterial hypertension) were more common in ischaemic aetiology of HF. Genetic testing did not identify pathogenic mutations in any of the patients, only variants of uncertain significance (Supplementary Table S1). Prior to the performed surgical procedures, all patients had high N-terminal pro-brain natriuretic peptide (NT-proBNP) concentrations and severe HF symptoms according to the New York Heart Association (NYHA) classification, whereas one patient was in cardiogenic shock. Echocardiography findings showed similar structural phenotypes associated with advanced HF (e.g., remodelled, dilated left ventricle (LV) with reduced ejection fraction (EF), enlarged right ventricle and high systolic pulmonary artery pressure), whereas speckle-tracking analysis provided insights into different functional phenotypes related to disease aetiology (Fig. 1).

**Table 1:** Patient characteristics.

	Apical coring samples (LVAD group)		Explanted hearts samples (Heart transplantation group)			
	DCM (LVAD)	ICM (LVAD)	DCM (HTx)	ICM-1 (HTx)	ICM-2 (HTx)	TCM (HTx)
General patient characteristics						
Age (years)	44	67	54	63	53	28
Sex	Male	Male	Male	Male	Male	Male
Body mass index (kg/m^2^)	24.3	39.7	25.6	30.1	29.4	30.8
Coronary artery disease	No	Yes	No	Yes	Yes	No
Diabetes mellitus	No	Yes	No	No	Yes	No
Arterial hypertension	No	Yes	No	No	Yes	No
Hyperlipidaemia	No	Yes	No	No	Yes	No
Atrial fibrillation	Yes	Yes	Yes	No	No	No
Ventricular tachycardia or fibrillation	Yes	No	No	No	Yes	No
Implanted device	CRT-D	ICD	CRT-D	No	ICD	No
NYHA class (I–IV)	III	IV	III	IV	III	III
INTERMACS class (I–VI)	5	3	3	1	6	3
Echocardiography parameters*						
LV ejection fraction (%)	30	35	25	40	30	10
LV end-diastolic diameter/volume (cm/ml)	6.4/198	6.8/279	7.4/214	5.4/105	7.3/235	7.8/270
Index left atrial volume (ml/m^2^)	51.9	33.6	85.2	58.3	68.8	43.4
RV end-diastolic diameter (cm)	3.8	3.7	4.6	5.1	3.9	5.0
TAPSE/RV fractional area change (mm/%)	16/25	22/32	15/20	8/14	17/32	8/50
Systolic pulmonary artery pressure (mmHg)	50	45	45	25	55	45
Laboratory parameters*						
NT-proBNP (pg/ml)	6514	4864	5959	1835	527	11,748
Estimated glomerular filtration rate (ml/min/1.73 m^2^)	> 60	> 60	> 60	> 60	> 60	> 60
Troponin T (ug/L)	69	77	17	109	10	–

*DCM* dilated cardiomyopathy, *ICM* ischaemic cardiomyopathy, *TCM* toxic cardiomyopathy, *LVAD* left ventricular assist device, *HTx* heart transplantation, *CRT-D* cardiac resynchronisation therapy—defibrillator, *ICD* implantable cardioverter defibrillator, *NYHA* New York Heart Association, *INTERMACS* interagency registry for mechanically assisted circulatory support, *LV* left ventricle, *RV* right ventricle, *TAPSE* tricuspid annular plane systolic excursion, *NTproBNP* N-terminal pro-brain natriuretic peptide.

*Parameters before treatment with LVAD or HTx.

### Three-dimensional tissue histopathology analysis

**Fig. 2:**
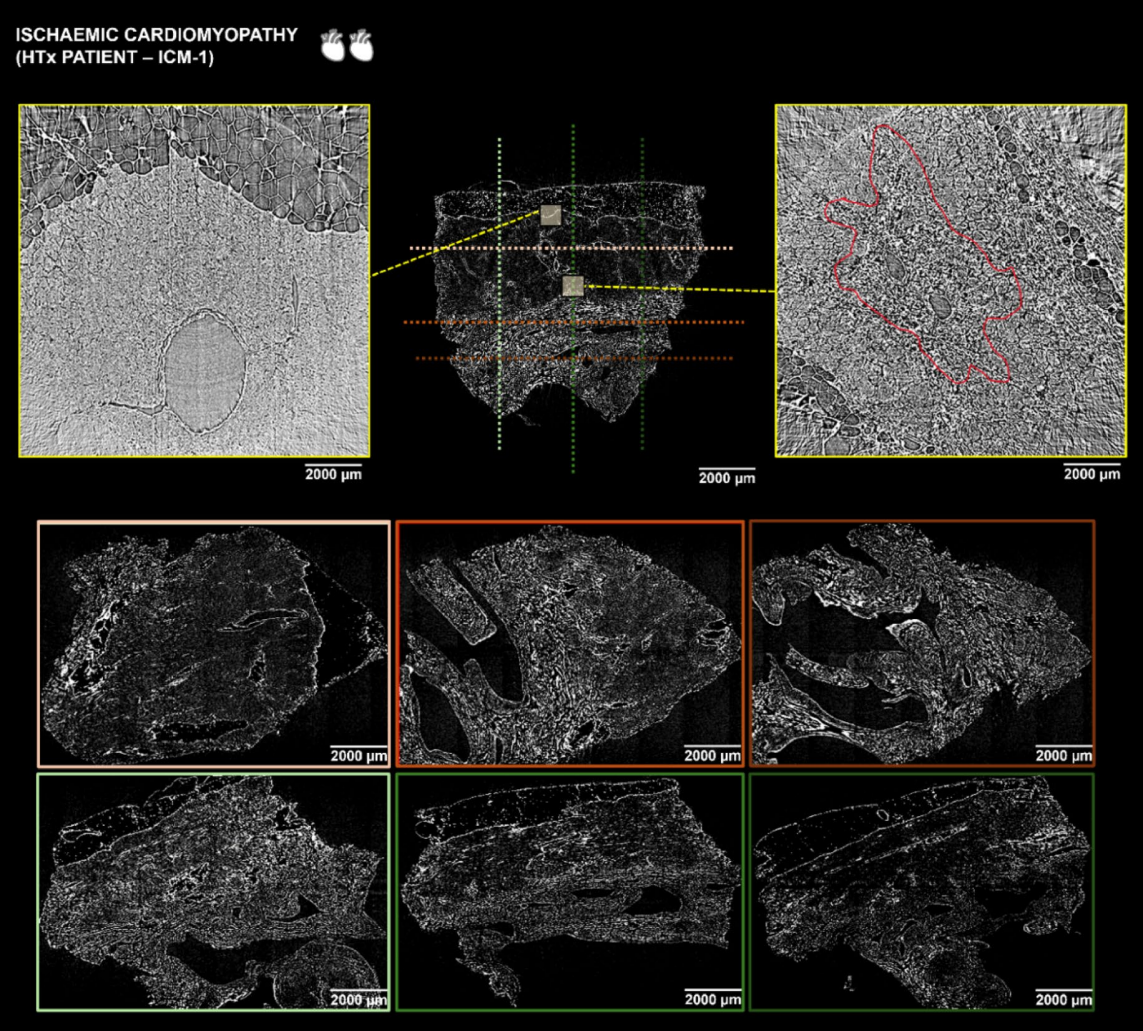
Virtual histopathology of ICM-1 (HTx) sample via X-PCI. Orthogonal views taken from a 3D X-PCI scan of the transmural myocardial tissue sample in a patient undergoing HTx due to ischaemic cardiomyopathy. The colour-coded horizontal and vertical dotted lines relate to the LR orthogonal cuts of the tissue sample shown in colour-coded frames below. The yellow rectangles show selected regions of the myocardium that were scanned with the HR imaging setup enabling analysis of cardiac microstructure. Fibrotic tissue replacement in the selected HR mid-myocardial region marked in red. Corresponding zoomed colour coded frames also shown in the Supplementary Fig. S1-S1.4. *ICM* ischaemic cardiomyopathy, *HTx* heart transplantation, *X-PCI* X-ray phase contrast imaging, *LR* low resolution, *HR* high resolution.

**Fig. 3:**
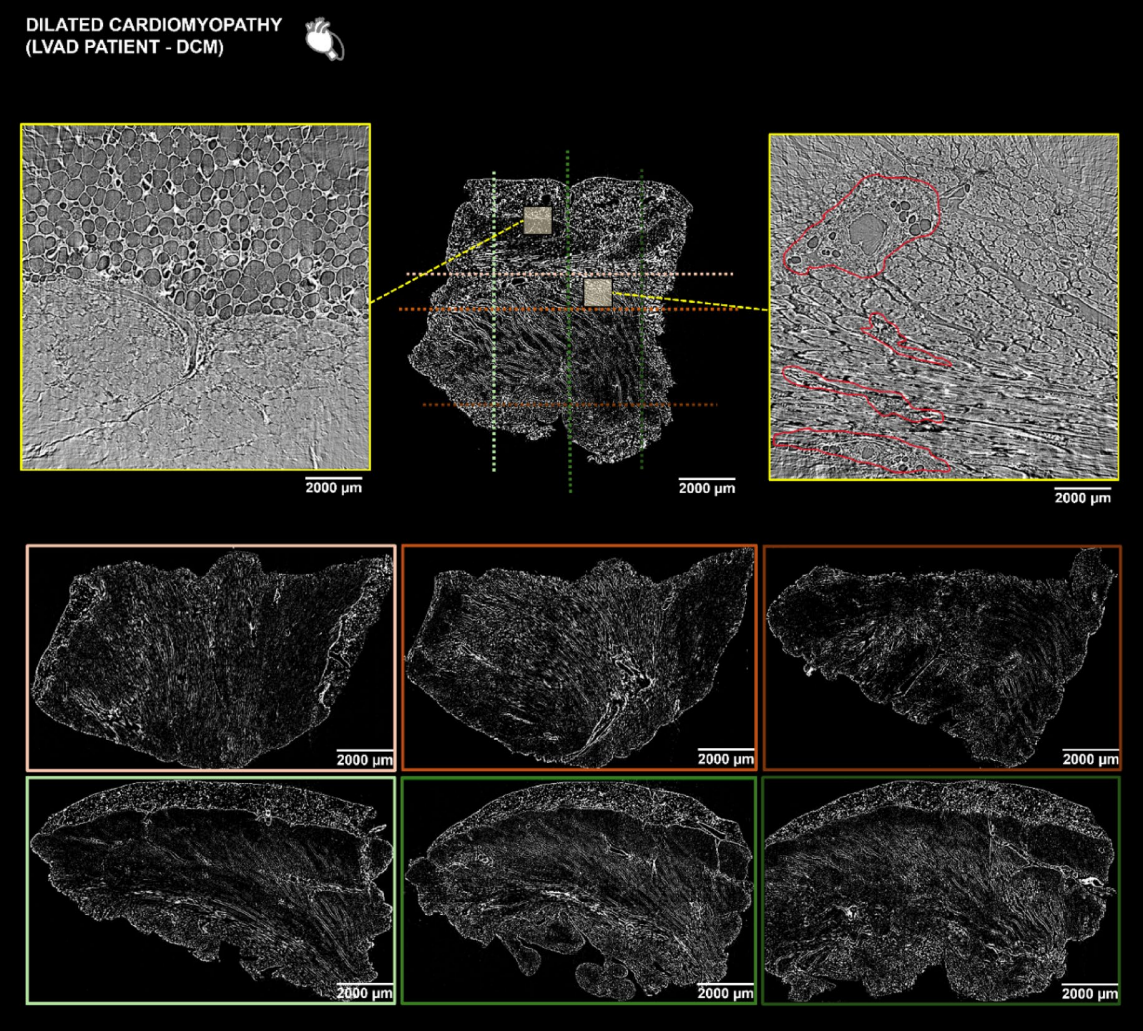
Virtual histopathology of DCM (LVAD) sample via X-PCI. Orthogonal views taken from a 3D X-PCI scan of the transmural myocardial tissue sample in a patient undergoing LVAD implantation due to dilated cardiomyopathy. The colour-coded horizontal and vertical dotted lines relate to the LR orthogonal cuts of the tissue sample shown in colour-coded frames below. The yellow rectangles show selected regions of the myocardium that were scanned with the HR imaging setup enabling analysis of cardiac microstructure. Perivascular and interstitial fibrotic changes in the selected HR mid-myocardial region marked in red. Corresponding zoomed colour coded frames also shown in the Supplementary Fig. S2-S2.4. *DCM* dilated cardiomyopathy, *LVAD* left ventricular assist device, *X-PCI* X-ray phase contrast imaging, *LR* low resolution, *HR* high resolution.

Image postprocessing included a 3D analysis of the low resolution (LR) X-PCI scan enabling a “virtual histopathology” of the full-thickness myocardial tissue (Figs. 2 and 3, Supplementary Figs. S1–S5). In the cross-sectional view of ICM-1 (heart transplantation (HTx)) data (Fig. 2 and Supplementary Fig. S1-S1.4) we could visualise interspersed perivascular and interstitial fibrosis and focal replacement of myocardium with scar tissue. On the other hand, dilated cardiomyopathy (DCM) (left ventricular assist device (LVAD)) cross-sectional slices (Fig. 3 and Supplementary Fig. S2-S2.4) showed enlarged cardiomyocytes with less extensive fibrotic changes around the vessels and in between cells. ICM (LVAD) and ICM-2 (HTx) transmural LR scans (Supplementary Figs. S3-S3.4 and S4-S4.4) showed similar morphological features as ICM-1 (HTx) with pronounced interstitial fibrosis and foci of scar tissue in the myocardium, whereas in TCM (HTx) (Supplementary Fig. S5-S5.4), there was significantly less fibrosis with foci of fatty tissue in the compact myocardium. The myocardium was further explored through the assessment of orientation of aggregates of myocytes.

### Quantification of cardiomyocyte aggregates’ orientation

**Fig. 4:**
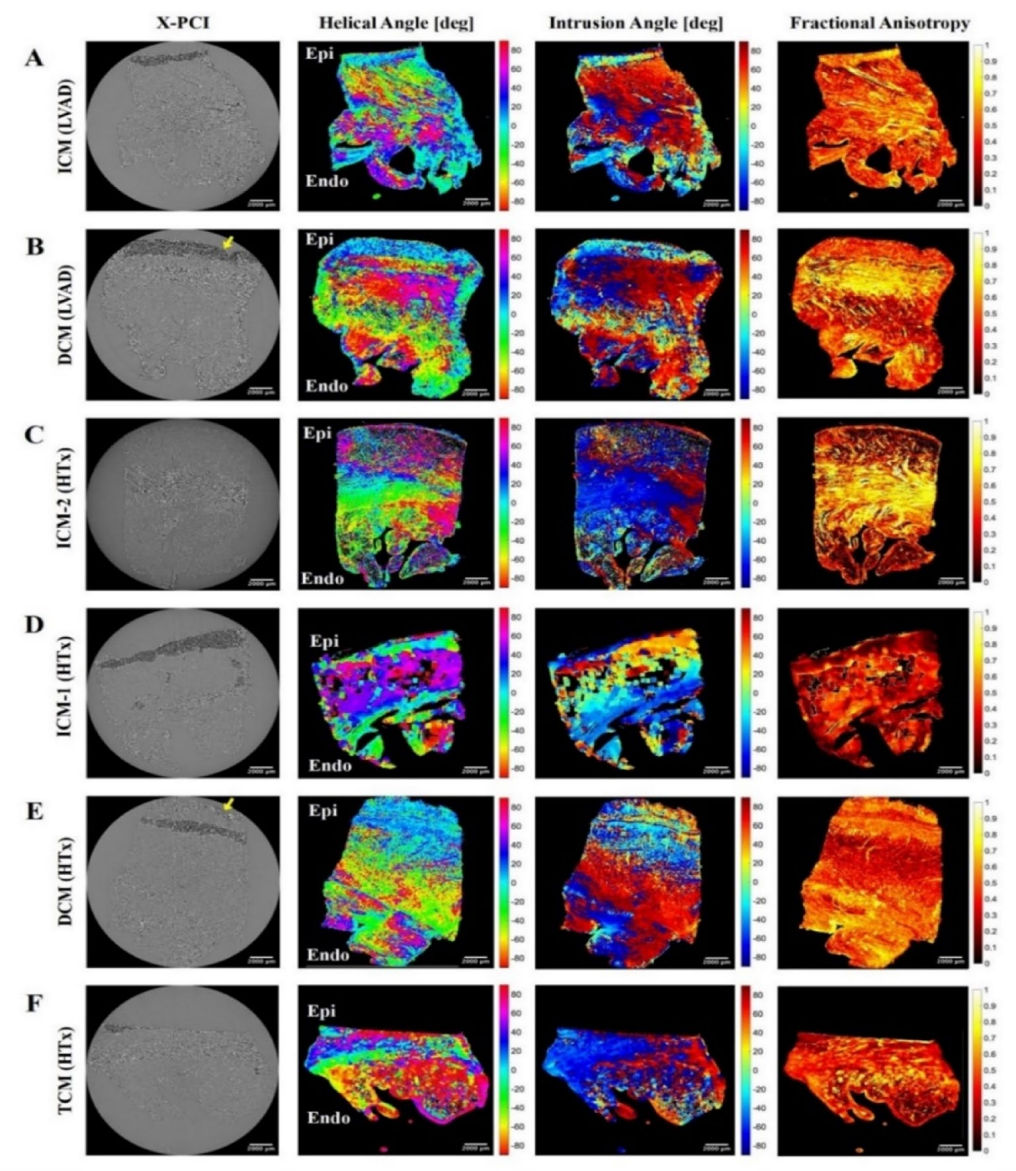
Visualisation of orientation of myocyte aggregates using helical angle (HA), intrusion angle (IA) and fractional anisotropy (FA). The epicardium and endocardium have been labelled as epi and endo, respectively. Epicardial adipose layer was thicker in DCM samples with a thinner epicardial myocyte layer (indicated by yellow arrows). *DCM* dilated cardiomyopathy, *ICM* ischaemic cardiomyopathy, *TCM* toxic cardiomyopathy, *LVAD* left ventricular assist device, *HTx* heart transplantation, *X-PCI* X-ray phase contrast imaging.

Visualisation of epicardial to endocardial transition of helical angle (HA) and intrusion angle (IA) in transmural samples showed a clear gradual transition in ICM-1 (HTx) and ICM-2 (HTx) samples (Fig. 4C,D). On the other hand, DCM samples lacked gradual transition and had a more abrupt pattern (Fig. 4B,E). ICM (LVAD), a sample obtained from apical coring, did not have a clear gradual transition compared to the other ICM samples. In all samples, the trabecular zone of the transmural sample was more disorganised in the compact myocardium as reflected in lower fractional anisotropy (FA) (Table 2) and higher IA (Fig. 4). A thin compact layer for TCM (HTx) could be observed relative to the trabecular region (Fig. 4F).

**Table 2:** Quantification of mean fractional anisotropy in whole myocardial tissue and percentage of collagen in selected region.

	Apical coring samples (LVAD group)		Explanted hearts samples (heart transplantation group)			
	DCM (LVAD)	ICM (LVAD)	DCM (HTx)	ICM-1 (HTx)	ICM-2 (HTx)	TCM (HTx)
Mean fractional anisotropy in selected area	0.5127	0.4915	0.4796	0.5454	0.3314	0.4179
Percentage of collagen in selected region (%)						
Sub-epicardium	2.72	10.35	2.57	1.57	5.36	1.37
Mid-myocardium	2.82	16.51	1.86	10.07	7.49	1.44
Sub-endocardium	1.99	27.41	2.46	11.01	4.43	0.87
Average	2.51	18.09	2.29	7.55	5.76	1.22

*DCM* dilated cardiomyopathy, *ICM* ischaemic cardiomyopathy, *TCM* toxic cardiomyopathy, *LVAD* left ventricular assist device.

### Semi-automated tissue collagen segmentation and quantification

**Fig. 5:**
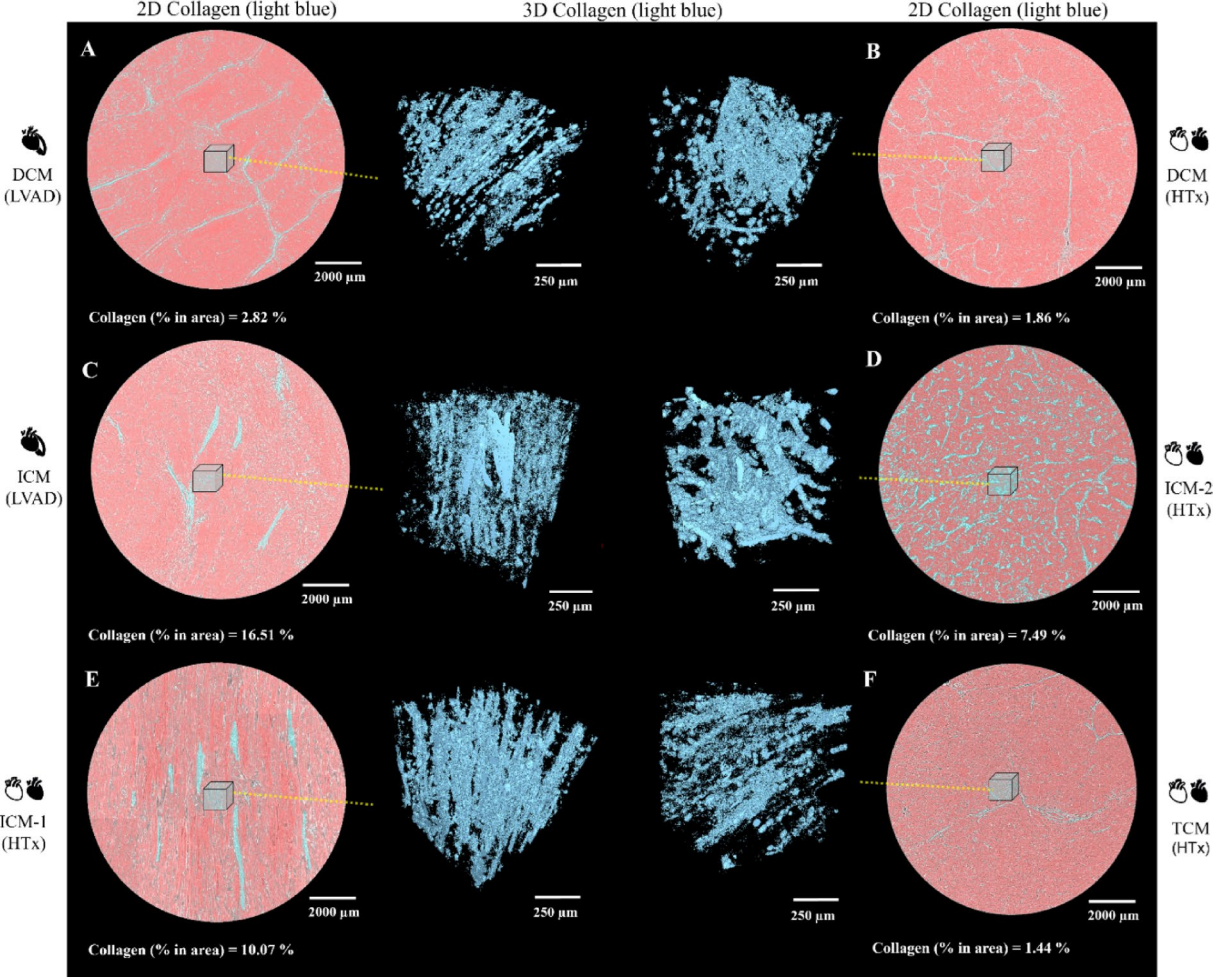
Semi-automatic 2D collagen segmentation and 3D rendering. Selected HR ROIs showing segmented collagen distribution in both 2D slices and 3D rendering. Collagen is shown in light blue, while myocardial cells are red in 2D slices. Rendered 3D models show just collagen in light blue. Light blue cubes represent the selected sub volume of tissue for the 3D models. Corresponding calculated percentages of collagen are shown below the selected 2D ROI. *HR* high resolution, *ROI* region of interest, *DCM* dilated cardiomyopathy, *ICM* ischaemic cardiomyopathy, *TCM* toxic cardiomyopathy, *HTx* heart transplantation, *LVAD* left ventricular assist device.

Semi-automatic two-dimensional (2D) collagen segmentation of the HR X-PCI data showed different patterns of collagen distribution. Both DCM samples showed diffuse, streaked pattern of thinner collagen fibrils spreading uniformly throughout the myocardium (Fig. 5A,B). On the contrary, ICM samples showed diffuse but thicker collagen fibres (located mostly in the subendocardial region), coupled with dispersed but transmural larger compact areas of collagen tissue (possible scar tissue as a sign of local ischemia) (Fig. 5C–E). The TCM myocardium had comparably less collagen fibrils but was interspersed with larger areas of adipose tissue surrounded by connective collagen matrix (Fig. 5F). Quantification of collagen proved a higher percentage of fibrosis in the selected regions of interest (ROI) for all the ICM samples compared to the DCM samples as well as the TCM sample (Fig. 5). Comparison of three selected ROI (sub-epicardium, mid-myocardium, sub-endocardium) showed uniform distribution of collagen throughout the DCM and TCM samples, whereas ICM samples had higher content of fibrosis on average, with ICM (LVAD) and ICM-1 (HTx) also showing more fibrosis in the subendocardial region (Table 2). Segmented data in the selected sub-volume of myocardial tissue was analysed and computationally processed to render a 3D model of collagen latticework spreading through the tissue, which showed fibre distribution and pattern in alignment with the 2D models (Fig. 5, middle images).

## Discussion

X-PCI has been previously used in visualisation and analysis of cardiac tissue micro- and macro-morphology in animal models of subclinical cardiac remodelling and myocardial infarction, as well as in distinguishing structural differences in patients with LV hypertrophy, showing various possibilities in structural exploration and quantification^10,12,13,15^. In this proof of principle study, we demonstrated the unique integration of 3D X-PCI with the standard diagnostic tools utilised in advanced HF with the goal of better understanding the underlying pathology and differentiation of cardiac disease.

The non-invasive imaging phenotype of advanced HF patients often overlaps in cardiomyopathies. In advanced HF the echocardiography phenotype often shows end-stage cardiac remodelling and chamber dilatation non-specific to the underlying disease (Fig. 1). While speckle tracking deformation analysis can help us differentiate aetiologies in LV hypertrophy or ischaemic disease (Fig. 1), there is little evidence for its use in differentiating dilated phenotypes. Cardiac magnetic resonance (CMR) can help with non-invasive tissue phenotyping. Nevertheless, patients referred for advanced HF treatment are often too sick for scanning or haemodynamically unstable, therefore unsuitable for a CMR imaging protocol (e.g., in our study one patient was in cardiogenic shock before HTx). Furthermore, a high percentage of chronic HF patients had a previously implanted pacemaker, implantable defibrillator or resynchronisation devices that reduce the possibility of high-quality tissue analysis (i.e., four out of six patients had a previously implanted device, hence most patients were not suitable candidates for a CMR scan). Finally, in cases where genetic disease is highly suspected, or when non-invasive imaging is inconclusive, genetic testing for pathogenic mutations is performed. Unfortunately, in the latter, results are often inconclusive or negative when tested for known mutations (such as the two DCM patients in our study).

In selected, diagnostically challenging cases where non-invasive imaging and genotyping do not provide a conclusive answer on aetiology, invasive imaging such as histopathology or X-PCI may bring important insights. Compared to histopathology, synchrotron-based X-PCI imaging overcomes the need for destructive tissue preparation steps (sample embedding, sectioning, and staining with contrast agents) and thus enables an unrestricted analysis of the whole, intact sample, enabling virtual slicing in all directions, and providing digital imaging datasets without any loss of information. The acquired X-PCI 3D datasets can then be further analysed via post-processing. In our cohort, the change in orientation of aggregates of myocytes from epicardium to endocardium was visualised and quantified, showing an aetiology-related disruption in the transition of the HA and IA from the epicardium to endocardium in DCM. In the DCM (LVAD) sample, a thick layer of epicardial fat is visible and disruption of the mid-myocardial layer is present (Fig. 4B). In the DCM (HTx) sample, disruption in myocyte orientation in the mid-compact zone could be observed (Fig. 4E). In the ICM (LVAD) tissue sample, we could visualise all myocardial layers without notable disruption besides high trabeculation (Fig. 4A).

Further analysis of the HR dataset enabled visualisation and quantification of fibrotic changes within the whole tissue sample, in 3D. ICM samples were characterised by the presence of higher collagen content (mostly in the subendocardial region) coupled with transmural scar formation (Fig. 5C–E). On the other hand, DCM samples showed a diffuse transmural fibrotic pattern of much lower percentage (Fig. 5A,B), reflecting changes that have previously been described in literature as structural differentiation^29,30^.

In the clinical setting, X-PCI analysis could improve data extraction and provide novel information as compared to conventional histopathology through non-destructive whole specimen analysis, myomapping and 3D analysis of patterns of fibrosis. Diseases with regional heterogeneity of microstructural changes or disruption of myocardial architecture (e.g., hypertrophic cardiomyopathy or DCM) might be optimal targets for such in-depth tissue analysis. As with any imaging modality, patient selection and cost-effectiveness is highly relevant. X-PCI as part of a multimodality imaging protocol could provide most benefit when phenotyping cardiac disease in selected patients with inconclusive findings from non-invasive imaging (due to disease stage, implanted devices, or poor image quality) and inconclusive genetic testing results, where determining diagnosis is clinically relevant due to prospective patient management or the risk of disease inheritance.

In conclusion, this proof of principle study has demonstrated the capability of synchrotron-based X-PCI for non-destructive advanced imaging of ex-vivo tissue, from the basic identification of tissue characteristics and histopathological changes directly from 3D datasets to quantitative assessment of orientation of aggregates of myocytes and collagen distribution. X-PCI can extend the amount of information available from ex-vivo tissue analysis, and, if used as an addition to multimodal imaging protocols in selected patients, potentially improve disease phenotyping and clinical diagnosis.

### Limitations

The analysed tissue samples were obtained by apical coring during LVAD implantation and after heart explanation from the anterolateral wall; therefore, the samples were potentially not representative of the whole heart. All included patients were male with either ICM, DCM or TCM, missing cardiac samples of hypertrophic or restrictive cardiomyopathy for a more comprehensive comparison. A larger and more diverse patient cohort would enable statistical analysis of established differences and the assessment of the reproducibility of findings.

Although the structure tensor is a useful method for estimating orientation of myocyte aggregates, it is sensitive to noise in the X-PCI datasets and the presence of artefacts (e.g., ring artefacts), and challenged by tissue regions with low grey level differences. When performing myomapping, only images with none or minimal artefacts were used, and a phase retrieval algorithm^31,32^ was applied to improve contrast and structure differentiation. Prospective technical development of the analysis methodology using additional appropriate filters for the structure tensor method^33^ will be discussed within the interdisciplinary study group and subsequently planned for future studies on expanded datasets.

Synchrotron-based X-PCI is currently employed at dedicated research facilities. Access is limited due to the scarcity of light sources available worldwide and the competitiveness in beamtime applications. This technique has already been used to visualise small cellular and subcellular features of cardiac samples^18,34,35^. Recently, with the development of new microfocus sources, dedicated acquisition strategies and algorithms, even propagation-based X-PCI is achievable on small samples at laboratory^36^. Those alternative approaches are currently being further developed to offer the possibility to integrate X-PCI into the clinical setting for improving clinical histopathology, diagnostics, and decision-making.

## Methods

### Patient data

The study included six patients with cardiomyopathies of different aetiology that were treated for advanced HF. Two patients received an LVAD, while four patients underwent HTx. Patients implanted with an LVAD (HeartMate 3, Abbott Laboratories, Chicago, Illinois, USA) were diagnosed with DCM and ICM, respectively. Patients undergoing HTx were diagnosed with ICM (two patients), DCM, and TCM. Data on demographic features, medical history, functional capacity (NYHA/INTERMACS class), cardiovascular risk factors, comorbidities (including hypertension, diabetes, metabolic syndrome), pharmacological treatment, and laboratory parameters (including blood count, kidney function, NT-proBNP, troponin) were collected. Genetic testing was performed based on a panel encompassing 174 genes, where the pathogenicity of variants was classified according to current recommendations^20^.

The study was conducted in accordance with the principles of the Declaration of Helsinki. Experiments were approved by the Ethical Committees of the University Hospital Centre Zagreb and University of Zagreb School of Medicine, both in Zagreb, Croatia. All patients provided written informed consent prior to enrollment. Tissues were procured via the Department of Cardiovascular Diseases at the University Hospital Centre Zagreb in Zagreb, Croatia. No tissues were procured from prisoners.

### Echocardiography

Transthoracic echocardiographic imaging was performed in all patients as a part of routine care, using the Vivid E95 system (GE Vingmed Ultrasound, Horten, Norway) equipped with a 4Vc transthoracic transducer. Full 2D and Doppler echocardiography were performed, while additional speckle tracking and Tissue doppler analysis was done in 4-chamber, 2-chamber, and 3-chamber apical acquisitions with appropriate frame rates, when possible. Chamber quantification was performed following the current recommendations^21^. LV function was assessed by EF and global longitudinal strain (GLS); right ventricular function was assessed through tricuspid annular plane systolic excursion (TAPSE) and the fractional area change (FAC).

### Synchrotron-based X-ray phase contrast imaging

Myocardial tissue samples were obtained either: (1) by apical coring during the LVAD implantation from the LV apical region, or (2) from the anterolateral wall in explanted hearts of HTx patients. Tissue samples were preserved in 10% buffered formaldehyde and transported to Paul Scherrer Institute (Villigen, Switzerland) where synchrotron-based X-PCI was performed at the TOMCAT X02DA beamline of the Swiss Light Source. An already established multi-scale X-PCI setup was used for image acquisition^22^. A LR configuration setup (5.8 µm pixel size) was used to capture overall morphology of the sample in correspondence with classical histopathology slides, as well as to estimate the orientation of myocyte aggregates. Regions of interest were selected from the obtained LR dataset to be imaged with a HR setup (0.65 µm pixel size), allowing for detailed visualisation of cardiomyocyte architecture, as well as for collagen segmentation.

### Imaging dataset analysis

Imaging datasets were processed in *Fiji* (ImageJ v.1.51 s, Wayne Rasband, National Institute of Health, USA)^23^ for visualisation and measurement of morphological features and structures. The 3D tissue scan was analysed by creating 2D orthogonal cuts in different myocardial layers. The entire 2D and 3D segmentation procedure was constructed in a step-by-step manner, and the results were evaluated by visual inspection and comparison between segmented and unprocessed data, in conjunction with a clinical pathologist specialising in cardiovascular pathology.

Quantification of orientation of myocyte aggregates, or ‘myomapping’, was performed on LR datasets using structure tensor analysis (an image analysis method that derives a tensor of image gradients in all directions within the neighbourhood of an image voxel) via an in-house MATLAB script (R2018b, MathWorks, Natick, MA, USA) as described previously^10–12,24^. A smoothing filter step was used before computing the image gradients and the same parameters (filter size of 123.5 × 123.5 × 123.5 µm) were used for all samples. Quantitative morphological parameters were extracted: helical angle, i.e., the angle between the circumferential left ventricular plane and myocyte aggregates; fractional anisotropy, i.e., the degree of anisotropy/disorganisation of the local myocardium; and intrusion angle, i.e., the angle between the local myocyte aggregate orientation and the radial direction of the ventricular wall (see Supplementary Fig. S6)^25,26^.

HR imaging datasets were used for visualisation, segmentation, and quantification of collagen, from which uniform regions that covered the sub-epicardium to sub-endocardium were selected for analysis. Collagen was segmented semi-automatically using a pixel classification workflow in the open-source software *Ilastik* (v.1.3.3, University of Heidelberg, Germany)^27^. From the resulting segmentation, the percentage of collagen present in the analysed volume was calculated using *Fiji* (ImageJ v.1.51 s, Wayne Rasband, National Institute of Health, USA). As a representation of a 3D collagen model, a uniform sub-volume of tissue in the form of a cube (voxel) was selected in the mid-myocardial region of each sample. The 3D collagen matrix model was visualised using the open-source software *Seg3D2* (Seg3D v. 2.2.1, University of Utah, USA)^28^. Additional information on data collection, genetic testing, image acquisition and image post-processing can be found in the Supplementary Methods.

## Electronic supplementary material

Below is the link to the electronic supplementary material.

Supplementary Material 1

## Data Availability

The datasets generated during and analysed during the current study are available from the corresponding author (maja.cikes@gmail.com) on reasonable request.
