# Supplementary material for: Synchrotron-based X-ray 3D phase contrast imaging and analysis of transmural myocardial tissue from heart failure patients
Source: Sci Rep. 2025 Jul 16;15:25867. doi: 10.1038/s41598-025-04012-5 (PMC12267503; doi:10.1038/s41598-025-04012-5)
Supplement: Supplementary file 1 — Supplementary Material 1 [file 41598_2025_4012_MOESM1_ESM.pdf]

Supplementary Data

Synchrotron-based X-ray 3D phase contrast imaging and analysis of transmural myocardial tissue from heart failure patients

Nikola Skreb[1†], Filip Loncaric[1†], Kan Yan Chloe Li[2†], Anne Bonnin[3], Hector Dejea[4], Patricia Garcia-Canadilla[5,6], Ivana Ilic[7], Hrvoje Gasparovic[8], Davor Milicic[1], Bart Bijnens[9,10], Andrew C Cook[2], Ivo Planinc[1#], Maja Cikes[1#*]

[1]University of Zagreb School of Medicine, Department of Cardiovascular Diseases, University Hospital Centre Zagreb, Zagreb, Croatia

[2]Institute of Cardiovascular Science, University College London, London, United Kingdom

[3]Swiss Light Source, Paul Scherrer Institute, Villigen, Switzerland

[4]European Synchrotron Radiation Facility, Grenoble, France

[5]Cardiovascular Diseases and Child Development, Sant Joan de Déu Research Institute (IRSJD), Esplugues de Llobregat, Spain

[6]Barcelona Centre for Maternal-Fetal and Neonatal Medicine (BCNatal), Hospital Sant Joan de Déu and Hospital Clínic, University of Barcelona, Barcelona, Spain

[7]University of Zagreb School of Medicine, Department of Pathology and Cytology, University Hospital Centre Zagreb, Zagreb, Croatia

[8]University of Zagreb School of Medicine, Department of Cardiac Surgery, University Hospital Centre Zagreb, Zagreb, Croatia

[9]Catalan Institution for Research and Advanced Studies, ICREA, Barcelona, Spain

[10]Universitat Pompeu Fabra, Barcelona, Spain

†Authors contributed equally as first authors.

#Authors contributed equally as senior authors.

Supplementary methods

Data collection

Cardiac tissue samples were placed in designated polystyrol tubes with adequate diameter fitting their size and filled with degassed deionised water. We used this medium for tissue structure preservation and reduction of bubble formation. Tubes were placed on the rotation stage where they were irradiated by a monochromatic, collimated X-ray beam with an energy of 20 keV. An established multi-scale X-PCI imaging setup was used, as described in Dejea [1]. Propagation-based (PB) phase contrast enhancement was achieved by placing the detector at a specific distance from the sample depending on the resolution. Low resolution (LR) datasets were acquired at 5.8 μm pixel size and high resolution (HR) datasets at 0.65 μm effective pixel size. The LR configuration setup had a sample-detector distance of 333 cm. A 1:1 microscope combined with a LuAG:Ce 300 μm scintillator (Crytur, Czech Republic) and a PCO.Edge 4.2 CMOS detector (PCO AG, Kelheim, Germany) was used to image the overall morphology of the sample, in correspondence with classical histology slides. Regions of interest (ROI) were selected from the obtained LR dataset to be imaged with a HR setup. This setup consisted of an X-ray microscope with a x10 magnification placed at 20 cm, combining a LuAG:Ce 20 μm scintillator (Crytur, Czech Republic) a x10 objective and a PCO.Edge 5.5 CMOS detector (PCO AG, Kelheim, Germany).

Imaging data reconstruction

The acquired projections were reconstructed using the Gridrec algorithm [2] both in absorption and applying the phase retrieval method by Paganin [3]. The δ/β value used for the Paganin method was finely tuned to 56.9. To cover the full sample in LR setting or the full ROI in HR, several overlapping scans were acquired and subsequently stitched together to obtain full LR and HR datasets for the analysis.

Quantification of orientation of aggregates of myocytes

To assess the orientation of myocyte aggregates, a structure tensor-based method was used via an in-house MATLAB script which has been fully described in previous published studies [4-7]. Central differences of pixel intensities were calculated in the three directions (x, y, z) of each

image voxel. The structure tensor was calculated at each voxel using prolate spheroidal coordinates (λ, μ, θ) for better representation of the elongated shape of myocyte aggregates than Cartesian coordinates [6]. Eigen decomposition of the structure tensor provided the three eigenvectors and eigenvalues. Eigenvalues represent magnitude of orientation of neighbouring cells in which the smallest (tertiary) eigenvector is considered the vector that aligns with the longitudinal axis of cardiomyocyte aggregates and corresponds with lowest intensity variation. To quantify myocardial organisation of the samples used in this study, the following morphologic parameters were computed: fractional anisotropy (FA), helical angle (HA), and intrusion angle (IA), also known as transverse angle (**Figure S4).**

Collagen segmentation and rendering

Imaging datasets were reviewed using the digital imaging tool *Fiji* (ImageJ v.1.51 s, Wayne Rasband, National Institute of Health, USA) [8], and from each sample HR dataset 250 equidistant images were selected (distance of 50 μm between images), with the aim of extending through the full depth of tissue transitioning from the epicardium, through myocardium, to the endocardium. Selected datasets were then processed and circularly cropped (to exclude imaging artefacts in the corners of the image) to 2000x2000 μm size for the purpose of further computational processing and analysis.

Collagen visualisation and segmentation in 2D was done with pixel classification workflow in *Ilastik* (v.1.3.3, University of Heidelberg, Germany) [9], a dedicated open-source software for image classification and segmentation. Colour labelling of collagen fibres, myocardial cells and extracellular space was used as an input for the semi-automatic segmentation algorithm application through the entire dataset. Walkthrough of the dedicated process in pixel classification workflow is shown in **Figure S5**. For the best computational performance and accuracy in segmentation, a sub-volume of tissue in the region of interest of the mid-myocardium was selected for each sample, forming a uniform voxel the size of 250x250x250 μm. Those selected voxels were then segmented for collagen in *Ilastik*, and the obtained segmentation results subsequently uploaded into a volume segmentation and image processing software *Seg3D2* (Seg3D v. 2.2.1, University of Utah, USA) [10], for the 3D rendering latticework model of collagen matrix.

Genetic testing

DNA used for the testing was isolated from peripheral whole blood samples using commercial kit for DNA isolation. Sequencing libraries were prepared with TruSight Cardio Sequencing panel (Illumina Inc., San Diego, USA). Sequencing was performed using MiniSeq System (Illumina Inc., San Diego, USA). Burrows-Wheeler Aligner (BWA) software package was used for sequence mapping, while Variant Interpreter software package (Illumina Inc., San Diego, USA) was used for data analysis and data visualization along with searching through Online Mendelian Inheritance in Man (OMIM) database, ClinVar archive, the Human Gene Mutation Database (HGMD) and database for single nucleotide polymorphisms (dbSNP). The panel encompassed 174 genes (its coding DNA sequences), with adequate gene coverage (>99% coverage at >20x). Pathogenicity of variants was classified according to current recommendations. Those variants considered clinically relevant according to the patient's phenotype were confirmed using Sanger sequencing technique. Genes included in cardiology panel are showed in **Table S1**.

Supplementary Figures

Supplementary Figure S1. Virtual histopathology of ICM-1 (HTx) sample via X-PCI. Orthogonal views taken from a 3D X-PCI scan of the transmural myocardial tissue sample in a patient undergoing HTx due to ischaemic cardiomyopathy. The yellow rectangles show selected regions of the myocardium that were scanned with the HR imaging setup enabling analysis of cardiac microstructure. Fibrotic tissue replacement in the selected HR mid-myocardial region marked in red. The colour-coded horizontal and vertical dotted lines relate to the LR orthogonal cuts of the tissue sample shown in zoomed colour-coded frames in the Supplementary figures S1.2-S1.4. below.

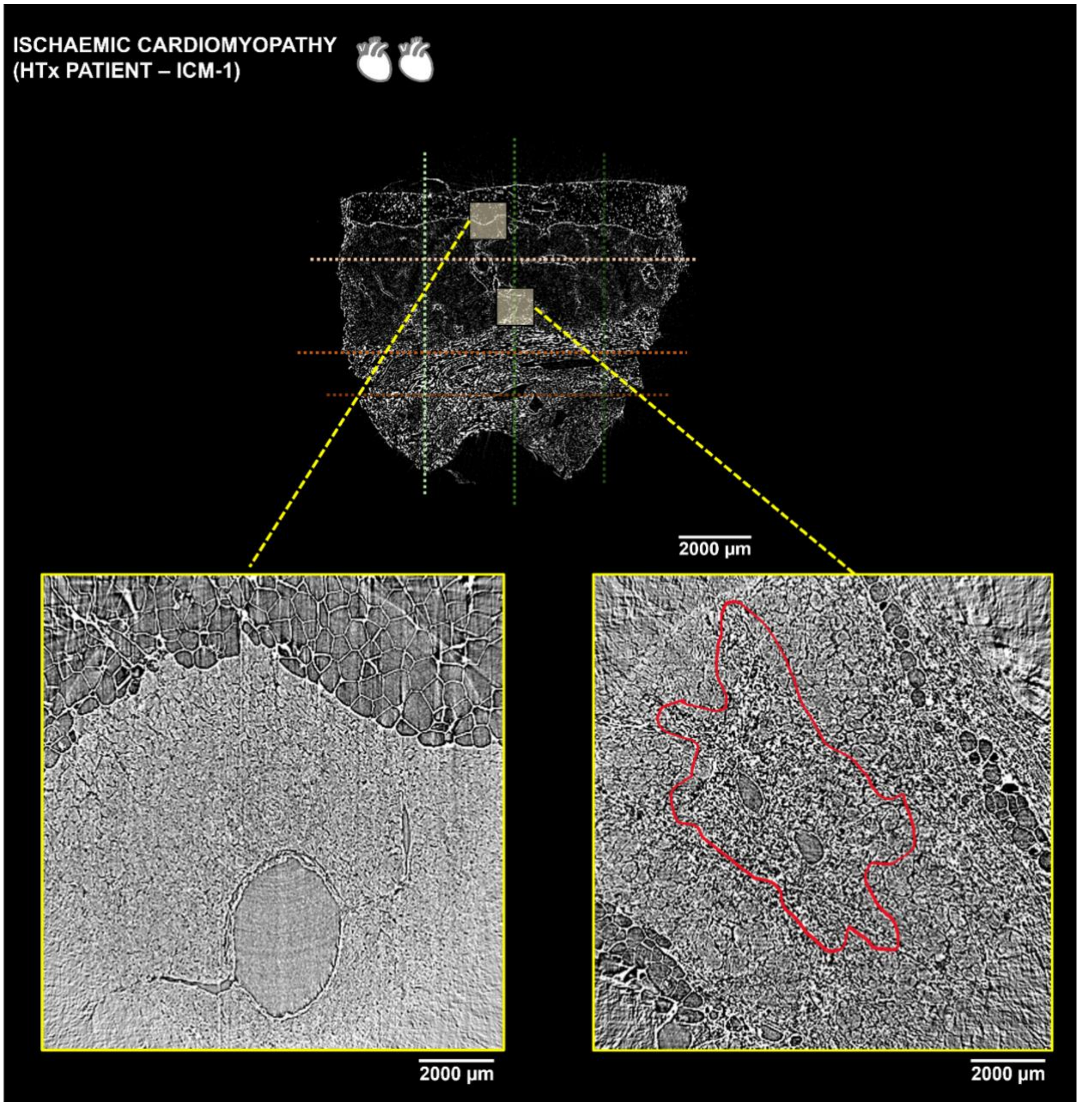

ICM=ischaemic cardiomyopathy; HTx=heart transplantation; X-PCI=X-ray phase contrast imaging; LR=low resolution; HR=high resolution

Supplementary Figure S1.2. Zoomed beige and orange colour-coded frames of LR orthogonal cuts of ICM-1 (HTx) sample.

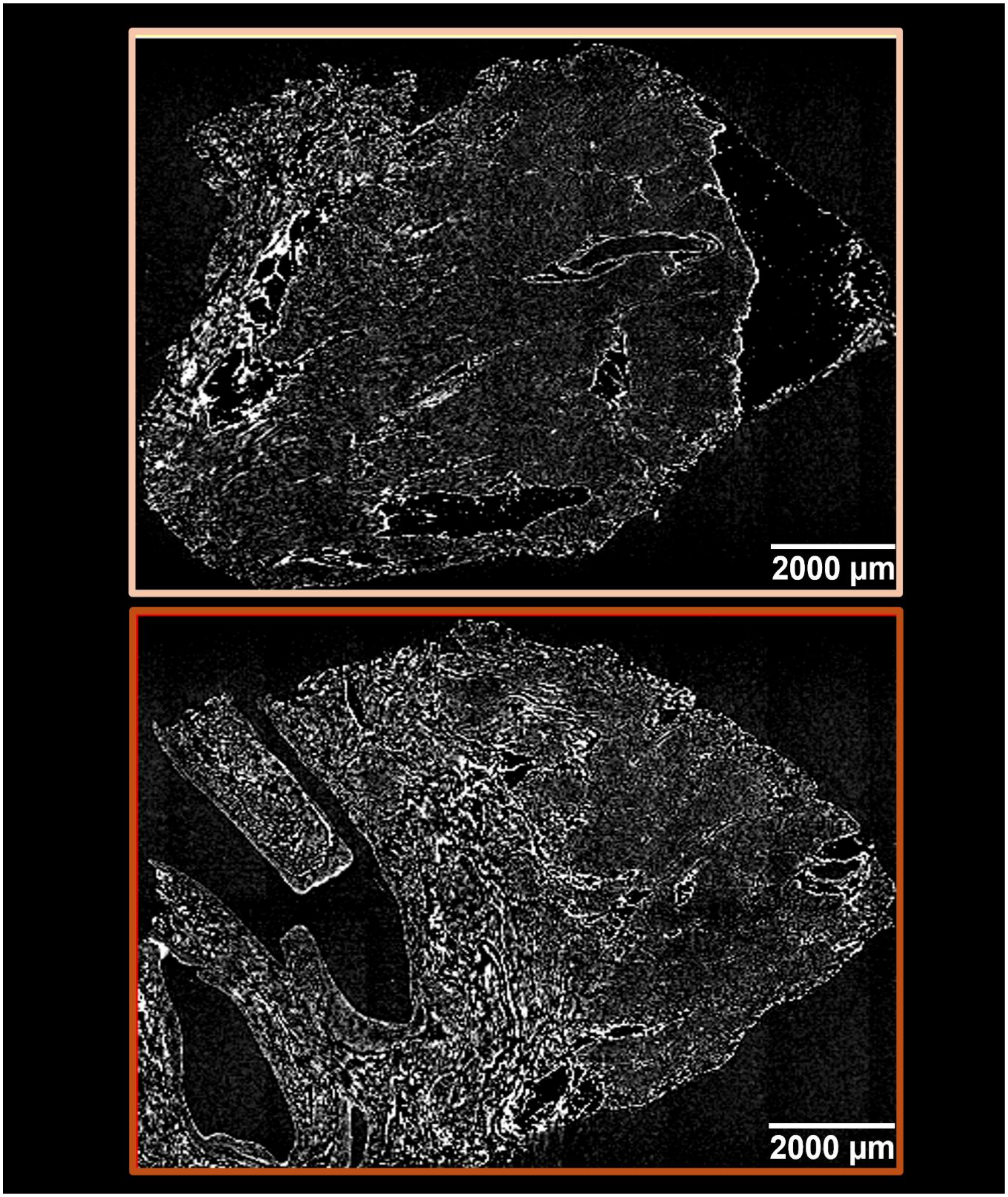

LR=low resolution; ICM=ischaemic cardiomyopathy; HTx=heart transplantation

Supplementary Figure S1.3. Zoomed brown and sage colour-coded frames of LR orthogonal cuts of ICM-1 (HTx) sample.

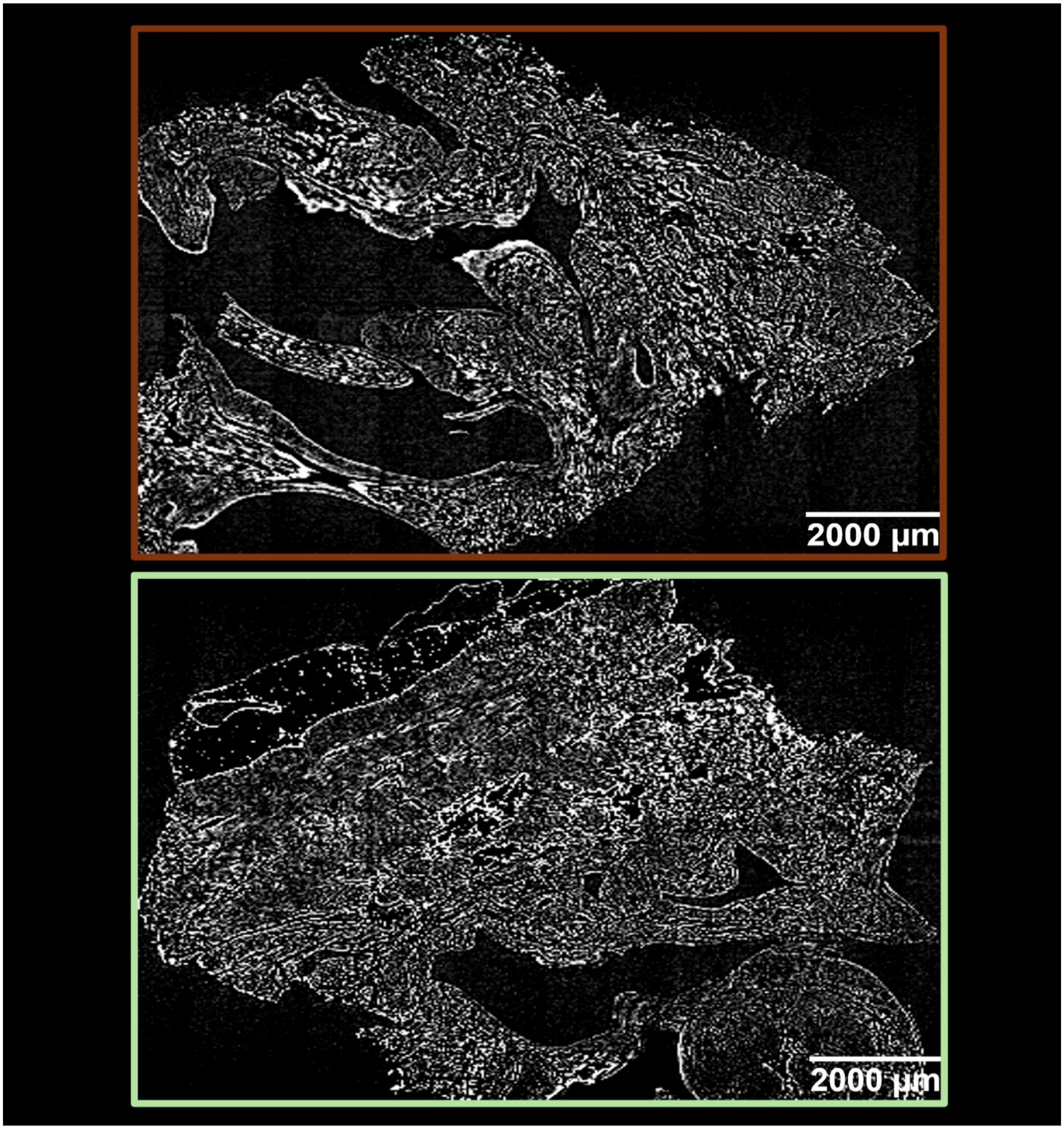

LR=low resolution; ICM=ischaemic cardiomyopathy; HTx=heart transplantation

Supplementary Figure S1.4. Zoomed green and dark green colour-coded frames of LR orthogonal cuts of ICM-1 (HTx) sample.

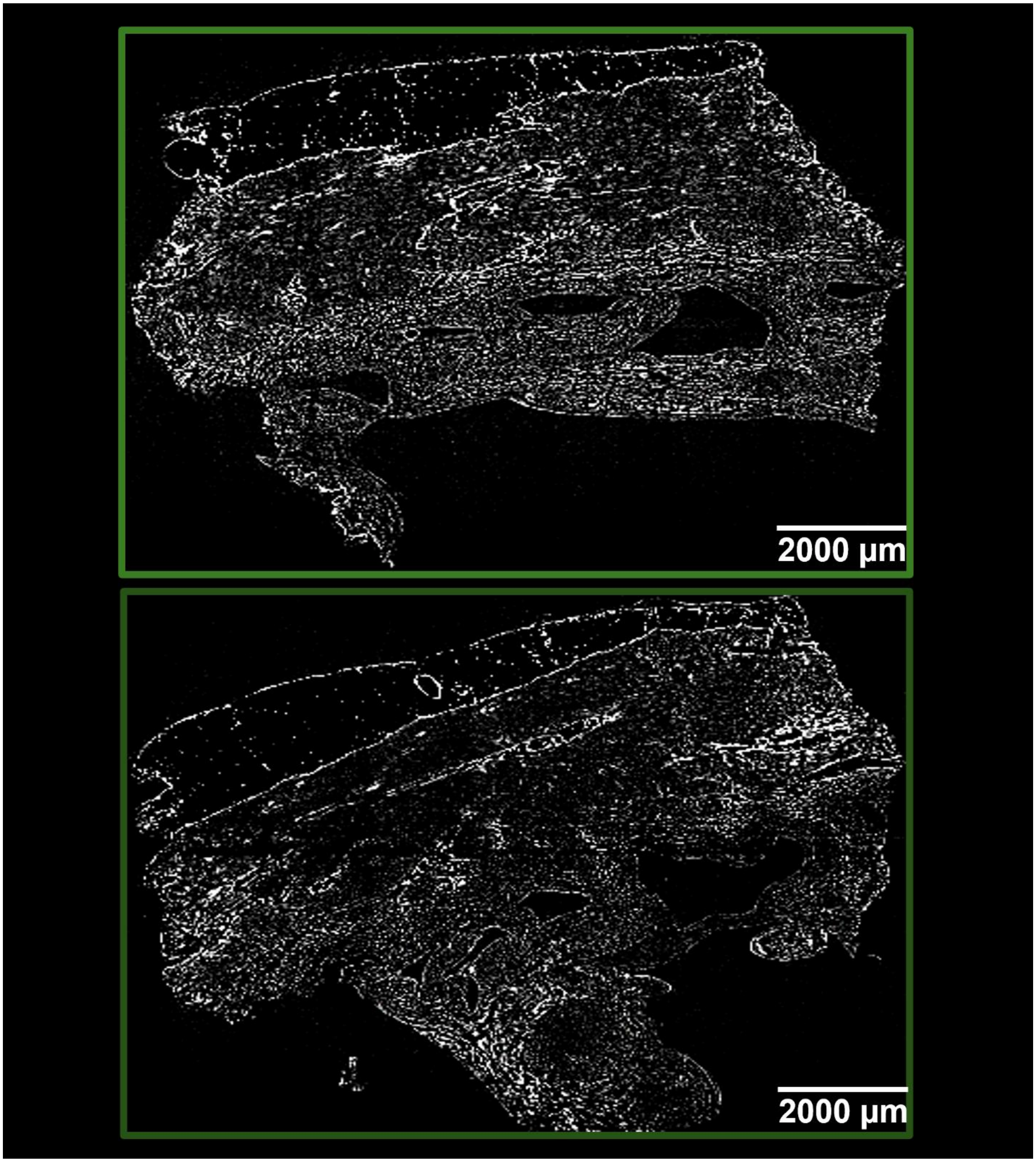

LR=low resolution; ICM=ischaemic cardiomyopathy; HTx=heart transplantation

Supplementary Figure S2. Virtual histopathology of DCM (LVAD) sample via X-PCI. Orthogonal views taken from a 3D X-PCI scan of the transmural myocardial tissue sample in a patient undergoing LVAD implantation due to dilated cardiomyopathy. The yellow rectangles show selected regions of the myocardium that were scanned with the HR imaging setup enabling analysis of cardiac microstructure. Perivascular and interstitial fibrotic changes in the selected HR mid-myocardial region marked in red. The colour-coded horizontal and vertical dotted lines relate to the LR orthogonal cuts of the tissue sample shown in zoomed colour-coded frames in the Supplementary figures S2.2-S2.4 below.

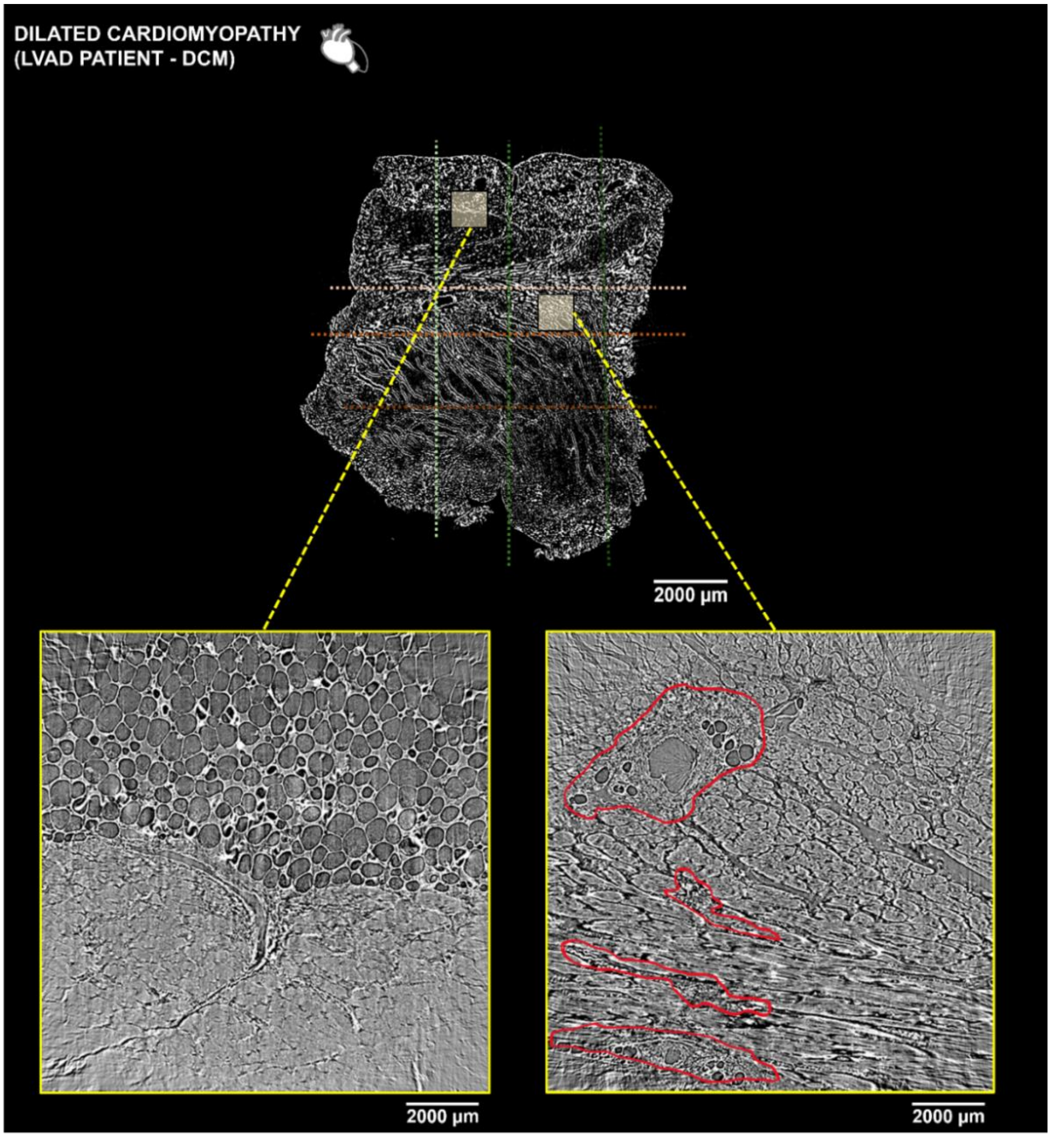

DCM=dilated cardiomyopathy; LVAD=left ventricular assist device; X-PCI=X-ray phase contrast imaging; LR=low resolution; HR=high resolution

Supplementary Figure S2.2. Zoomed beige and orange colour-coded frames of LR orthogonal cuts of DCM (LVAD) sample.

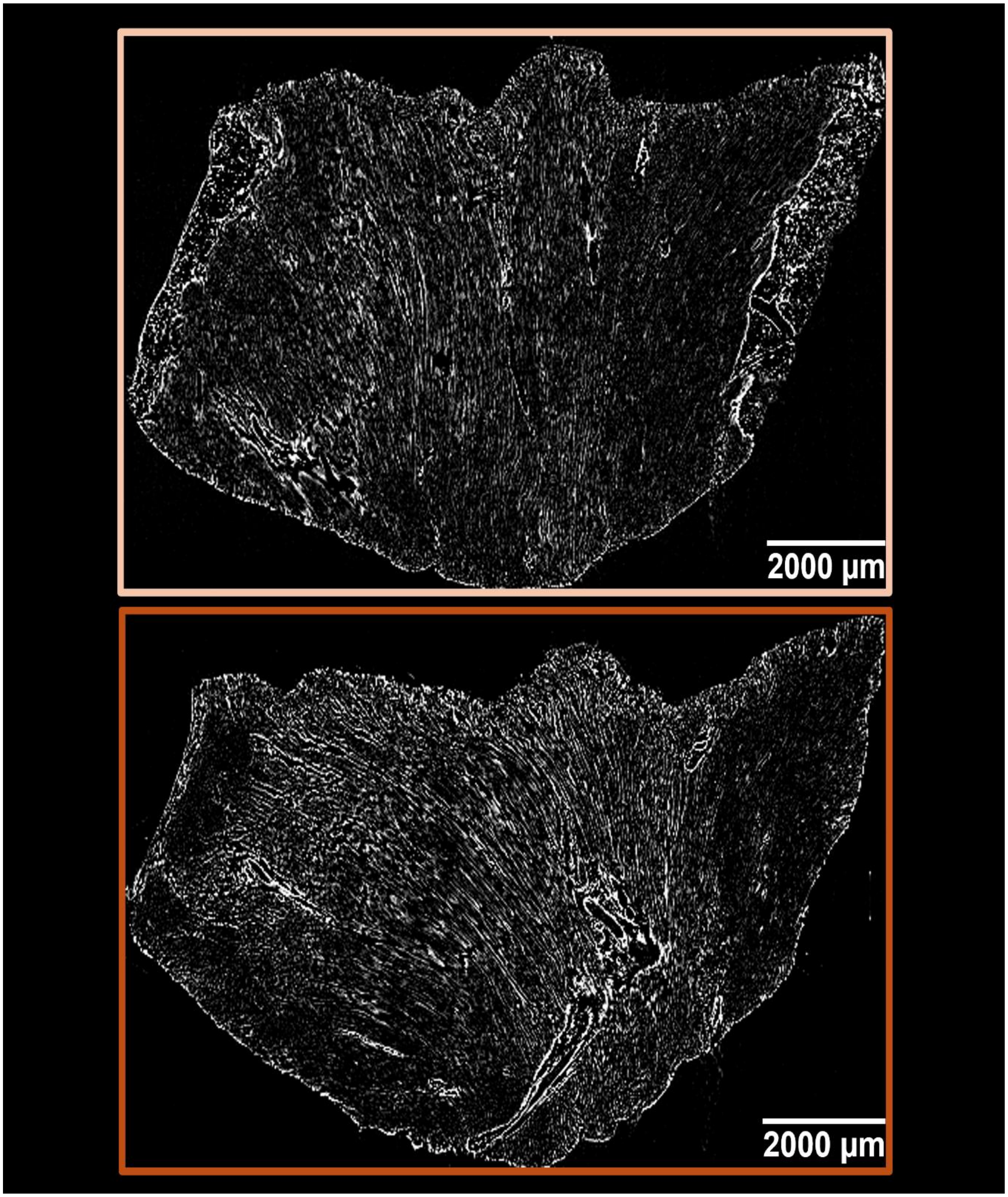

LR=low resolution; DCM=dilated cardiomyopathy; LVAD=left ventricular assist device

Supplementary Figure S2.3. Zoomed brown and sage colour-coded frames of LR orthogonal cuts of DCM (LVAD) sample.

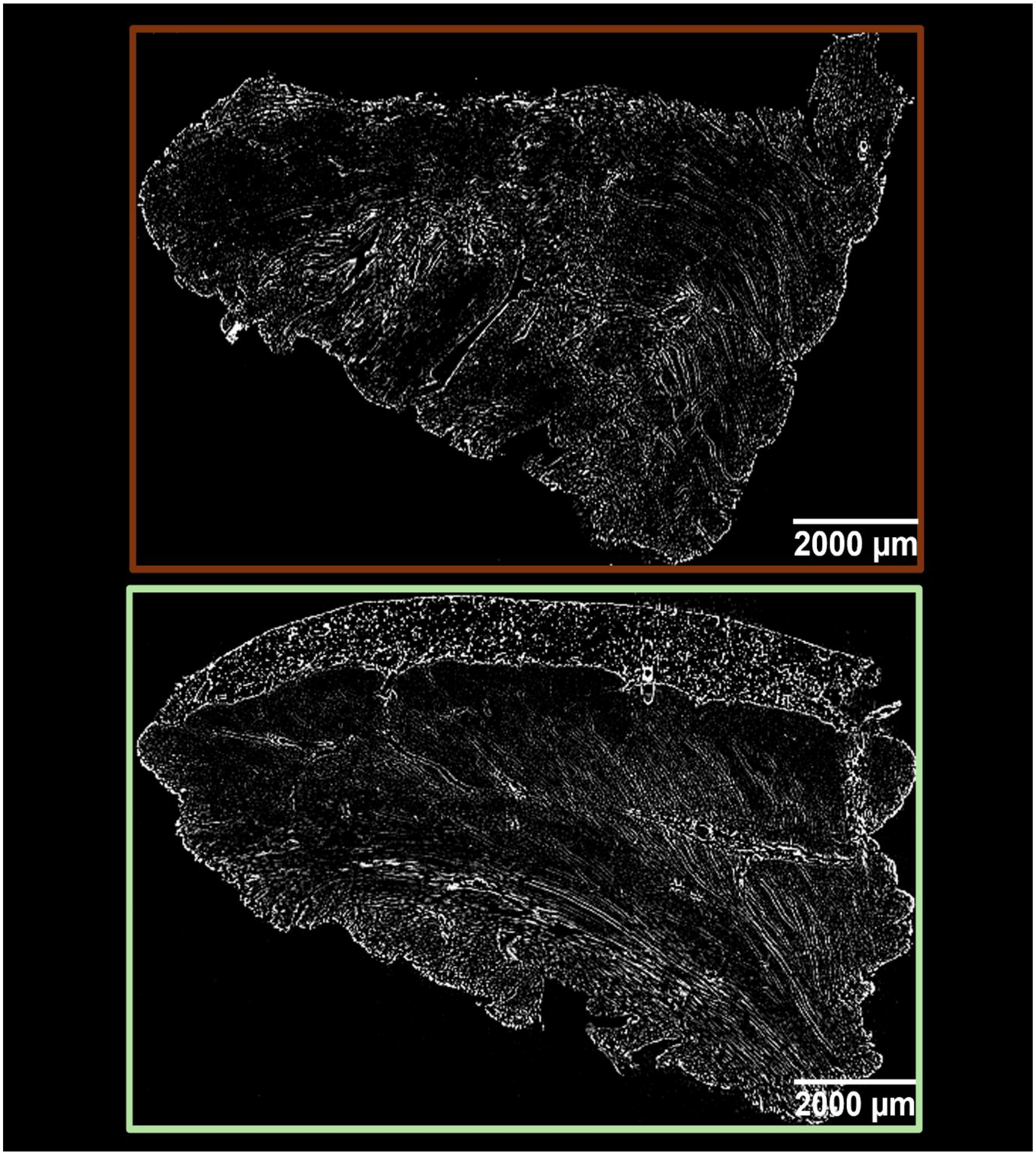

LR=low resolution; DCM=dilated cardiomyopathy; LVAD=left ventricular assist device

Supplementary Figure S2.4. Zoomed green and dark green colour-coded frames of LR orthogonal cuts of DCM (LVAD) sample.

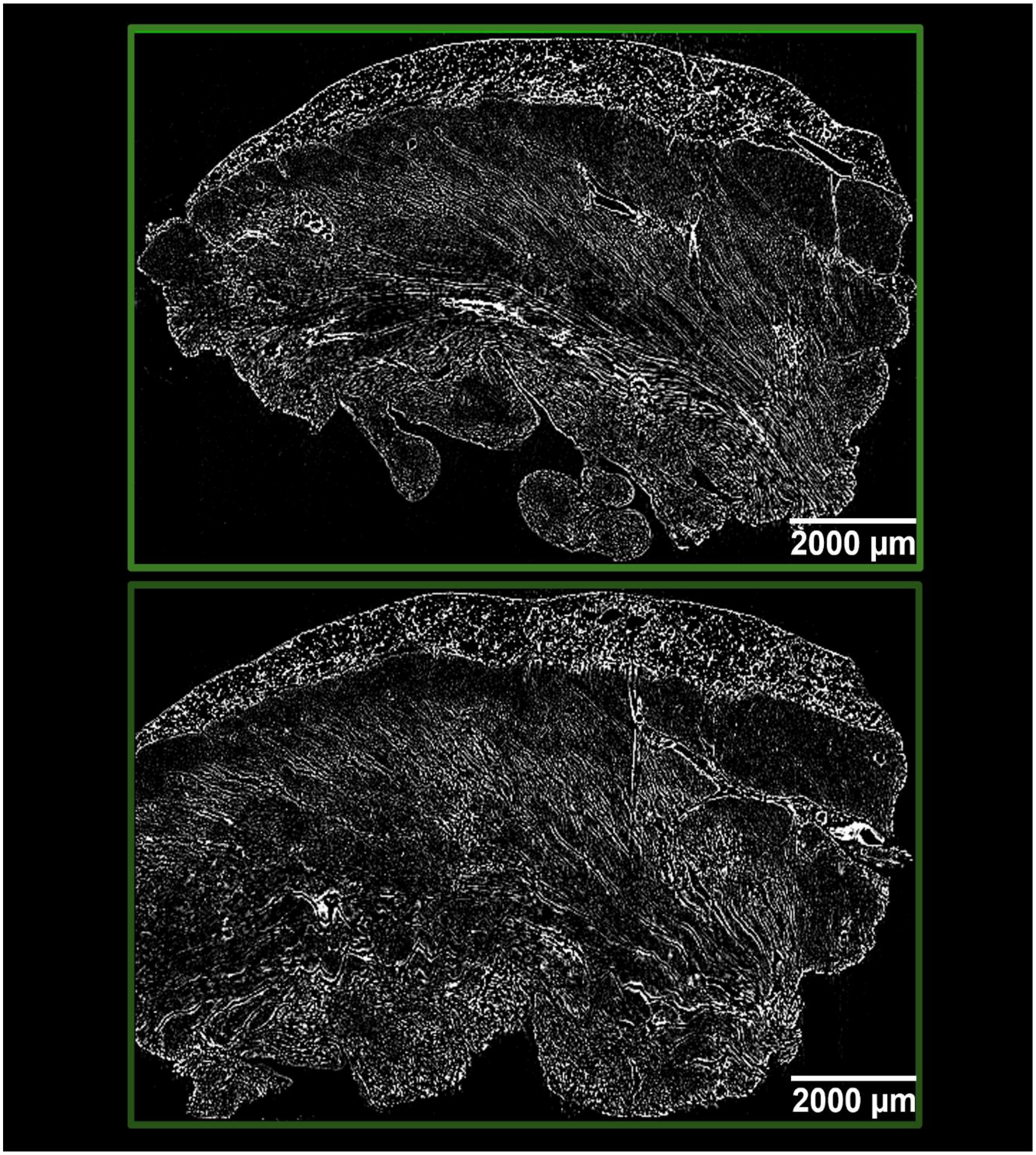

LR=low resolution; DCM=dilated cardiomyopathy; LVAD=left ventricular assist device

Supplementary Figure S3. Virtual histopathology of ICM-2 (HTx) sample via X-PCI. Orthogonal views taken from a 3D X-PCI scan of the transmural myocardial tissue sample in a patient undergoing HTx due to ischaemic cardiomyopathy. The yellow rectangles show selected regions of the myocardium that were scanned with the HR imaging setup enabling analysis of cardiac microstructure. Interstitial fibrotic changes in the selected HR mid-myocardial region marked in red. The colour-coded horizontal and vertical dotted lines relate to the LR orthogonal cuts of the tissue sample shown in zoomed colour-coded frames in the Supplementary figures S3.2-S3.4 below.

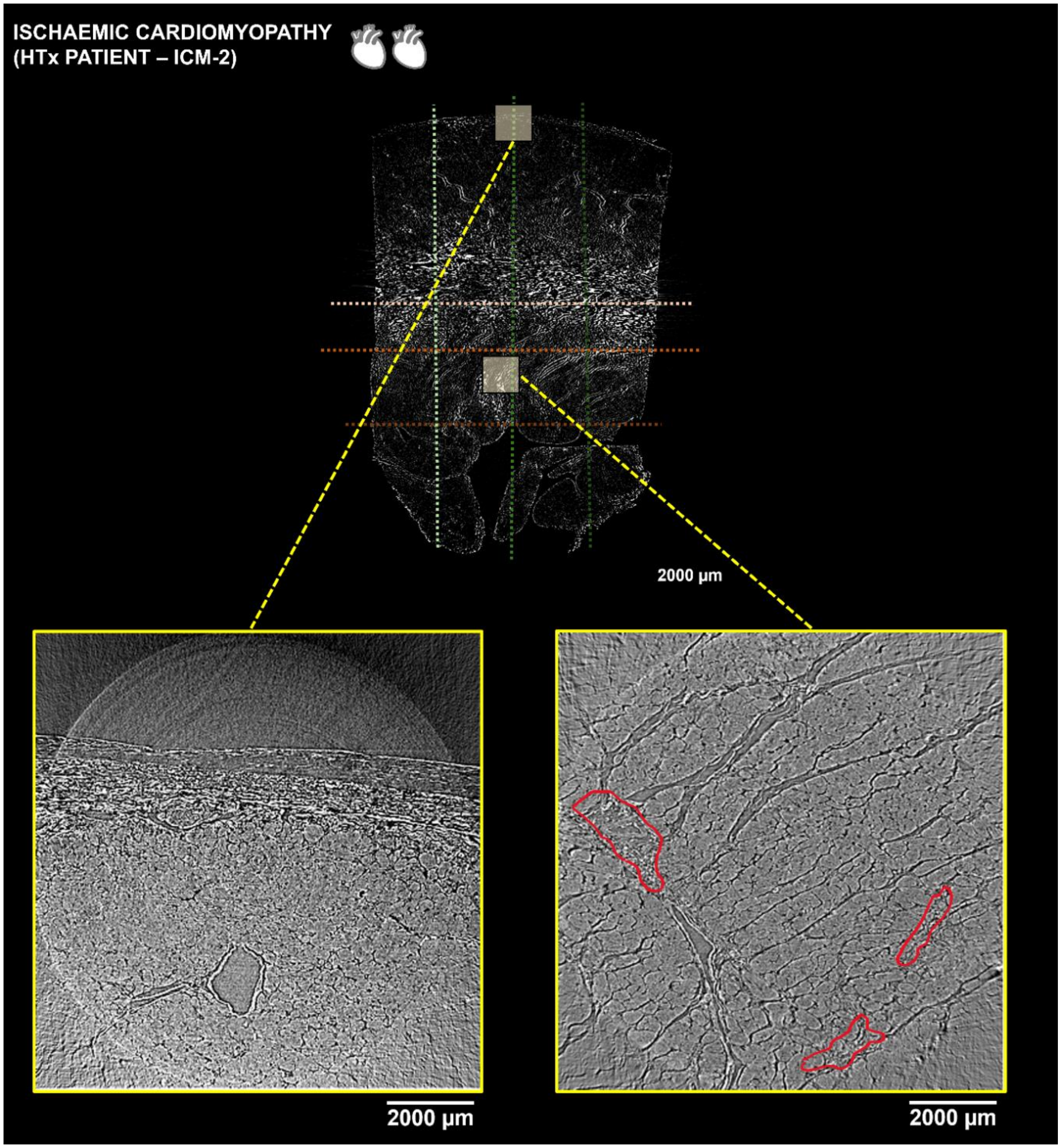

ICM=ischaemic cardiomyopathy; HTx=heart transplantation; X-PCI=X-ray phase contrast imaging; LR=low resolution; HR=high resolution

Supplementary Figure S3.2. Zoomed beige and orange colour-coded frames of LR orthogonal cuts of ICM-2 (HTx) sample.

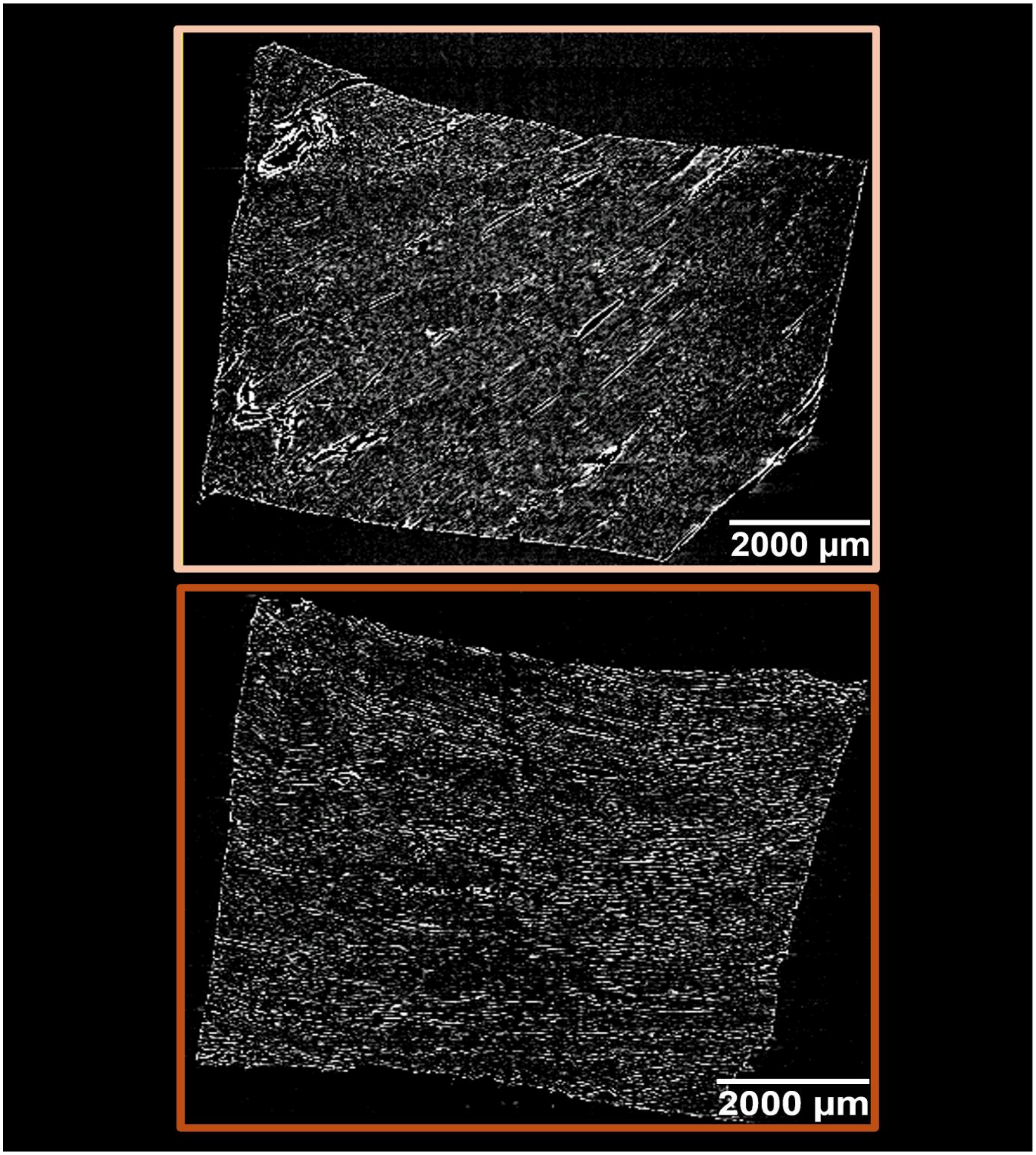

LR=low resolution; ICM=ischaemic cardiomyopathy; HTx=heart transplantation

Supplementary Figure S3.3. Zoomed brown and sage colour-coded frames of LR orthogonal cuts of ICM-2 (HTx) sample.

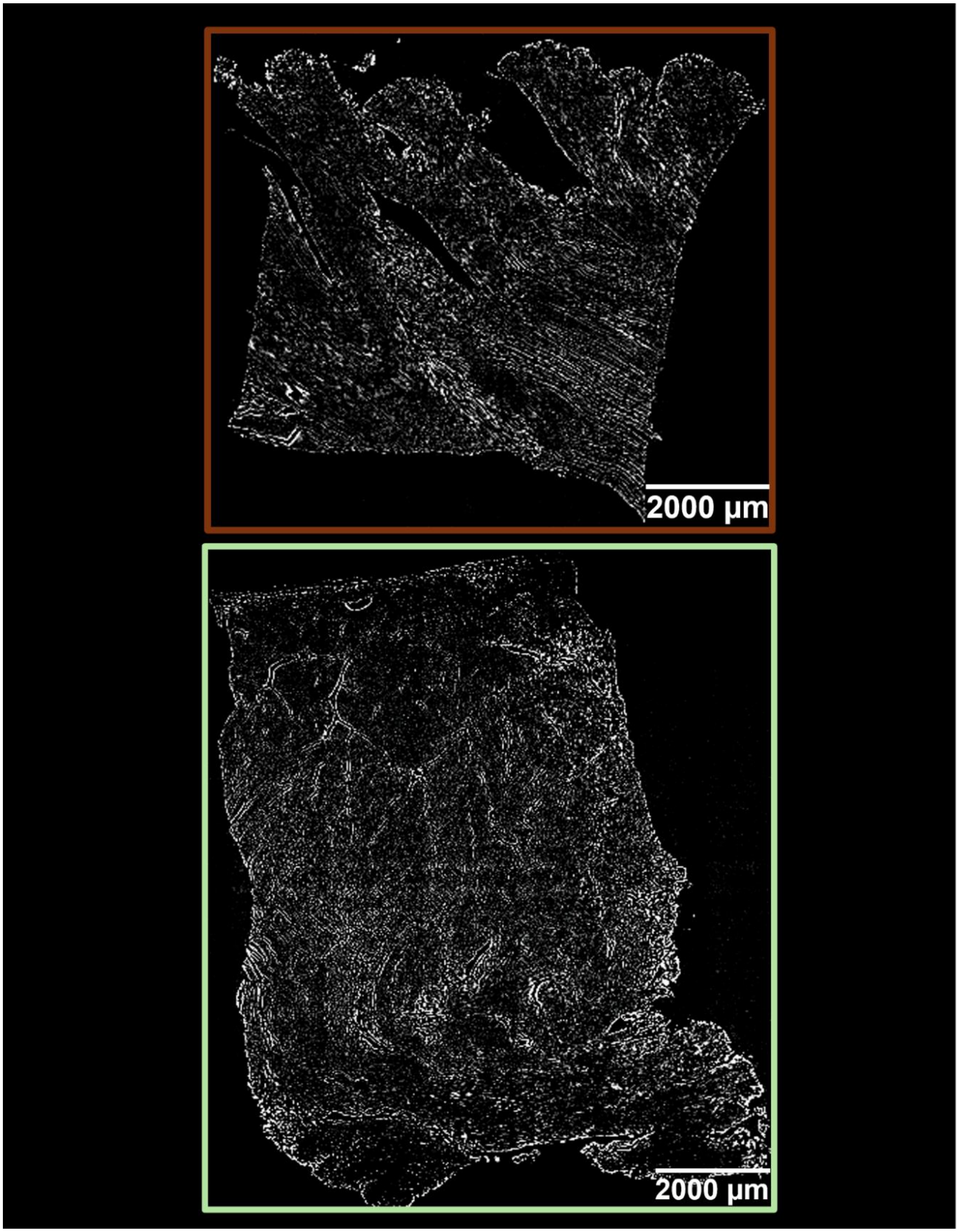

LR=low resolution; ICM=ischaemic cardiomyopathy; HTx=heart transplantation

Supplementary Figure S3.4. Zoomed green and dark green colour-coded frames of LR orthogonal cuts of ICM-2 (HTx) sample.

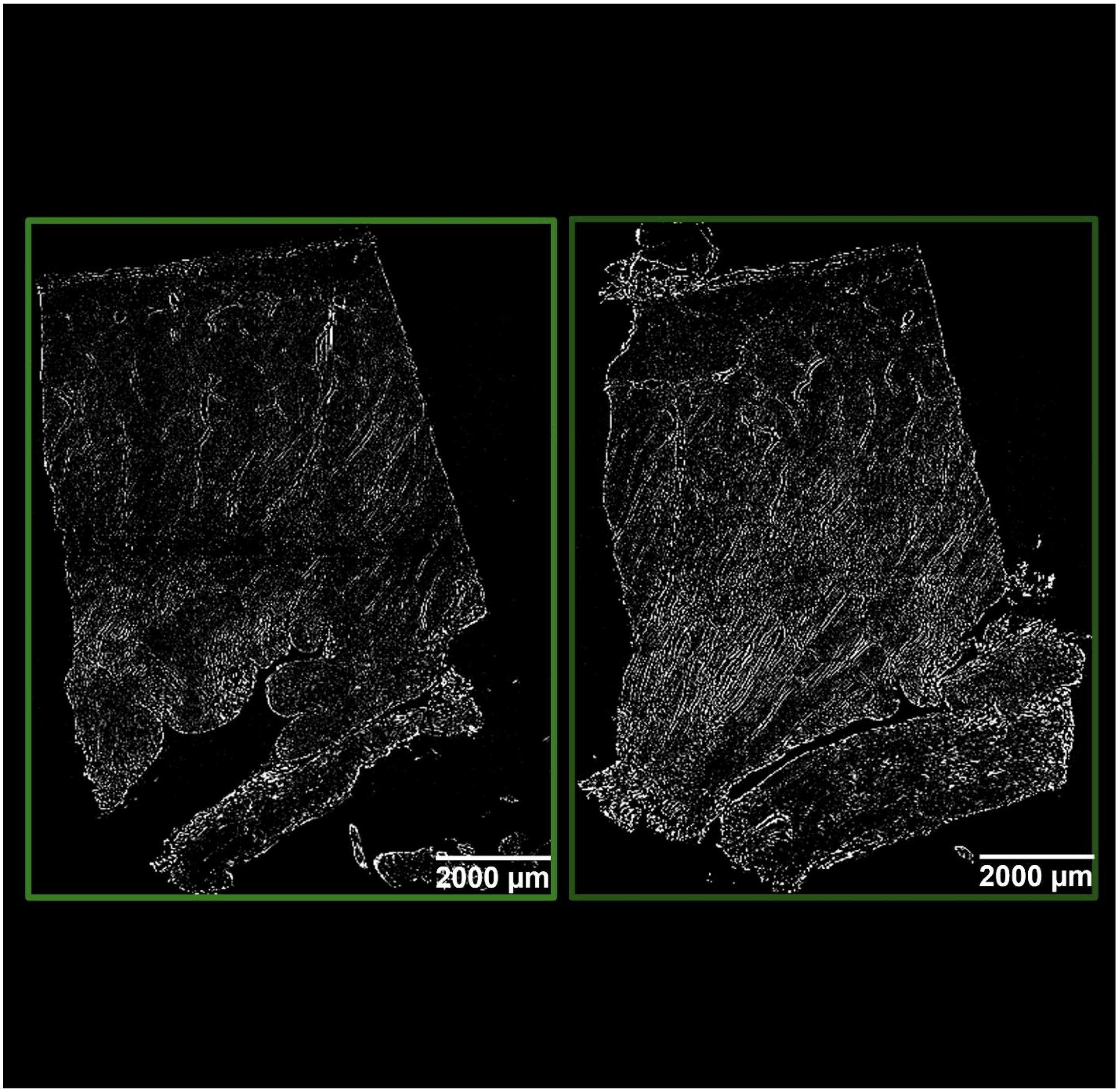

LR=low resolution; ICM=ischaemic cardiomyopathy; HTx=heart transplantation

Supplementary Figure S4. Virtual histopathology of ICM (LVAD) sample via X-PCI. Orthogonal views taken from a 3D X-PCI scan of the transmural myocardial tissue sample in a patient undergoing LVAD implantation due to ischaemic cardiomyopathy. The yellow rectangles show selected regions of the myocardium that were scanned with the HR imaging setup enabling analysis of cardiac microstructure. Interstitial fibrotic changes in the selected HR mid-myocardial region marked in red. The colour-coded horizontal and vertical dotted lines relate to the LR orthogonal cuts of the tissue sample shown in zoomed colour-coded frames in the Supplementary figures S4.2-S4.4 below.

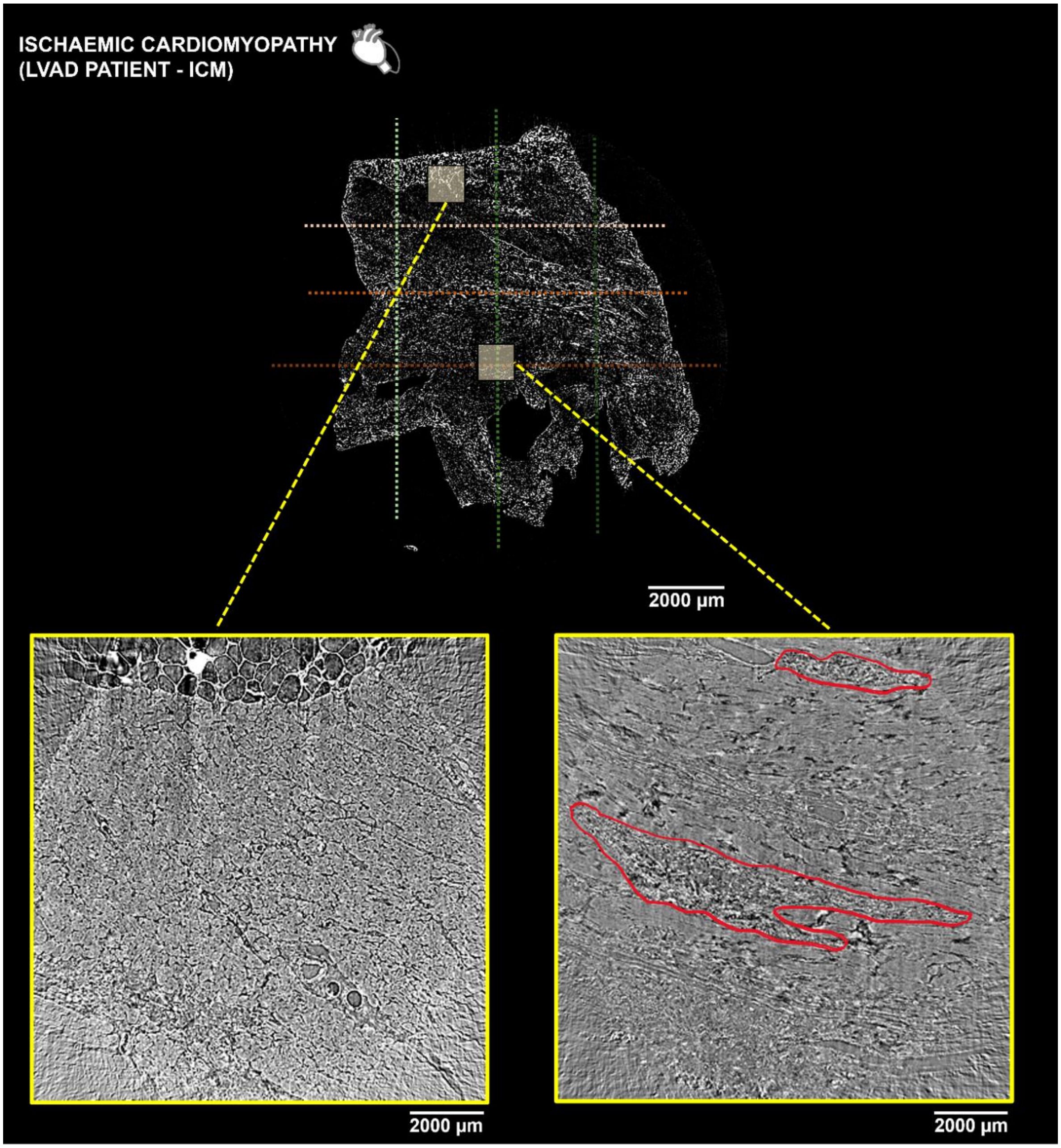

ICM=ischaemic cardiomyopathy; LVAD=left ventricular assist device; X-PCI=X-ray phase contrast imaging; LR=low resolution; HR=high resolution

Supplementary Figure S4.2. Zoomed beige and orange colour-coded frames of LR orthogonal cuts of ICM (LVAD) sample.

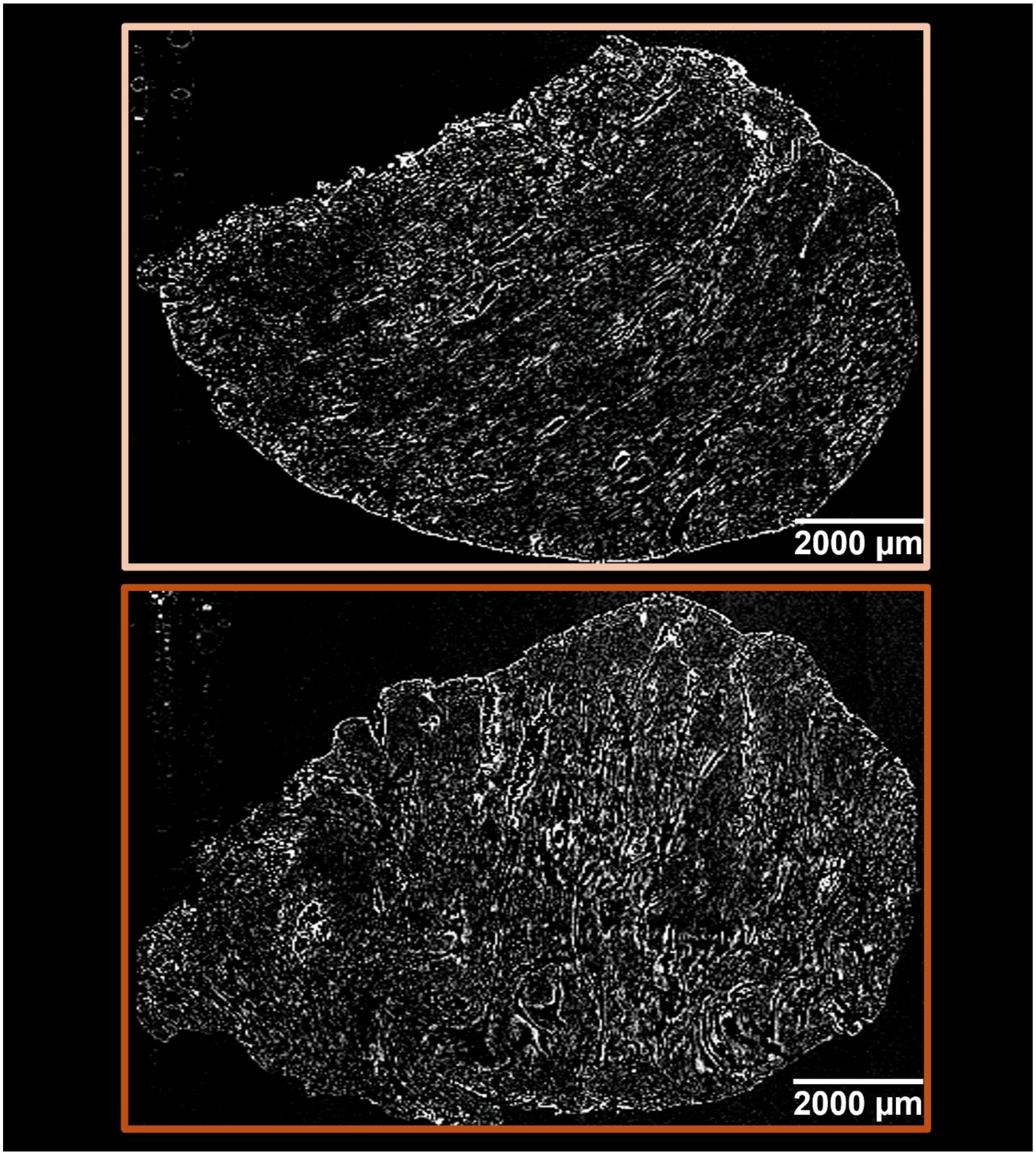

LR=low resolution; ICM=ischaemic cardiomyopathy; LVAD=left ventricular assist device

Supplementary Figure S4.3. Zoomed brown and sage colour-coded frames of LR orthogonal cuts of ICM (LVAD) sample.

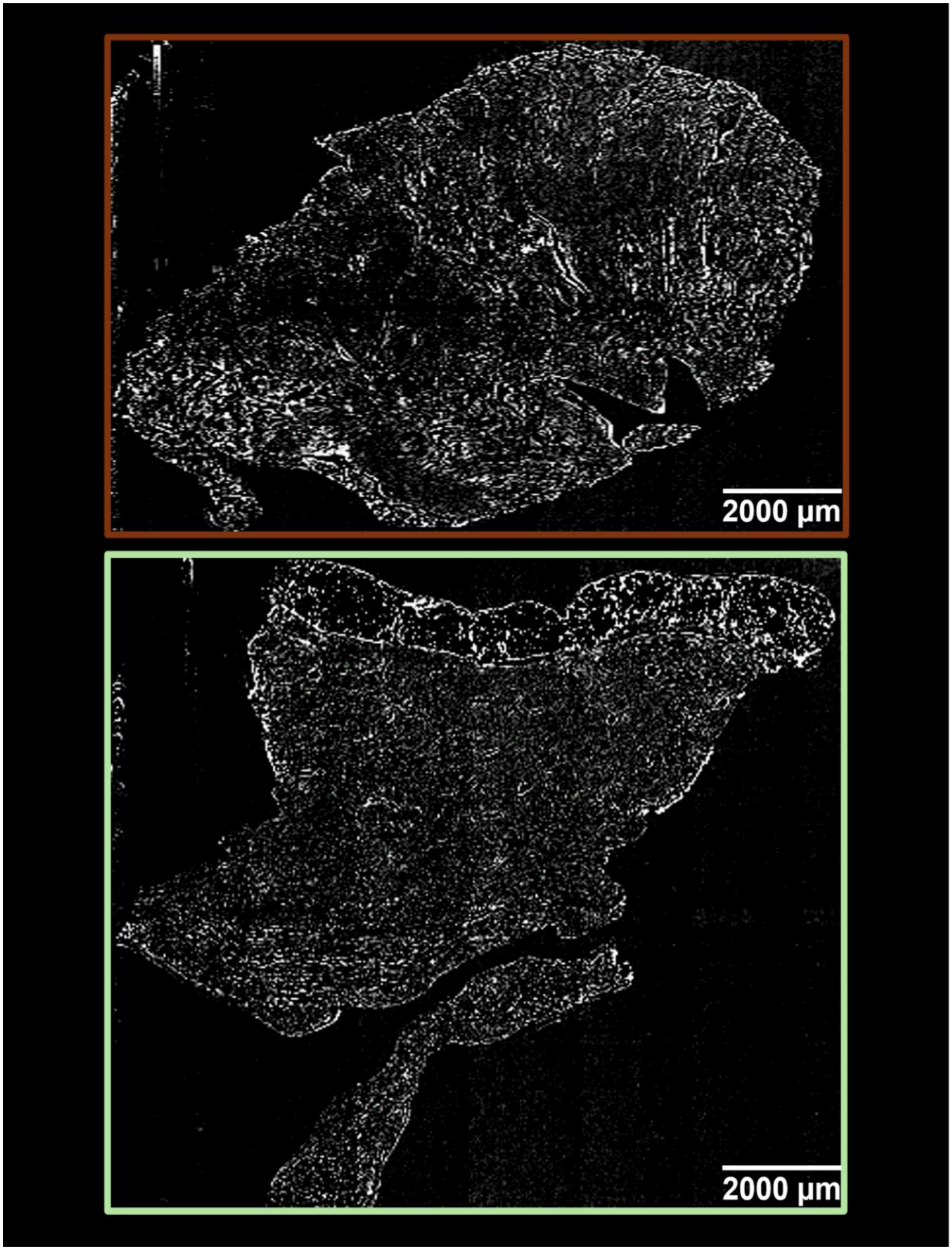

LR=low resolution; ICM=ischaemic cardiomyopathy; LVAD=left ventricular assist device

Supplementary Figure S4.4. Zoomed green and dark green colour-coded frames of LR orthogonal cuts of ICM (LVAD) sample.

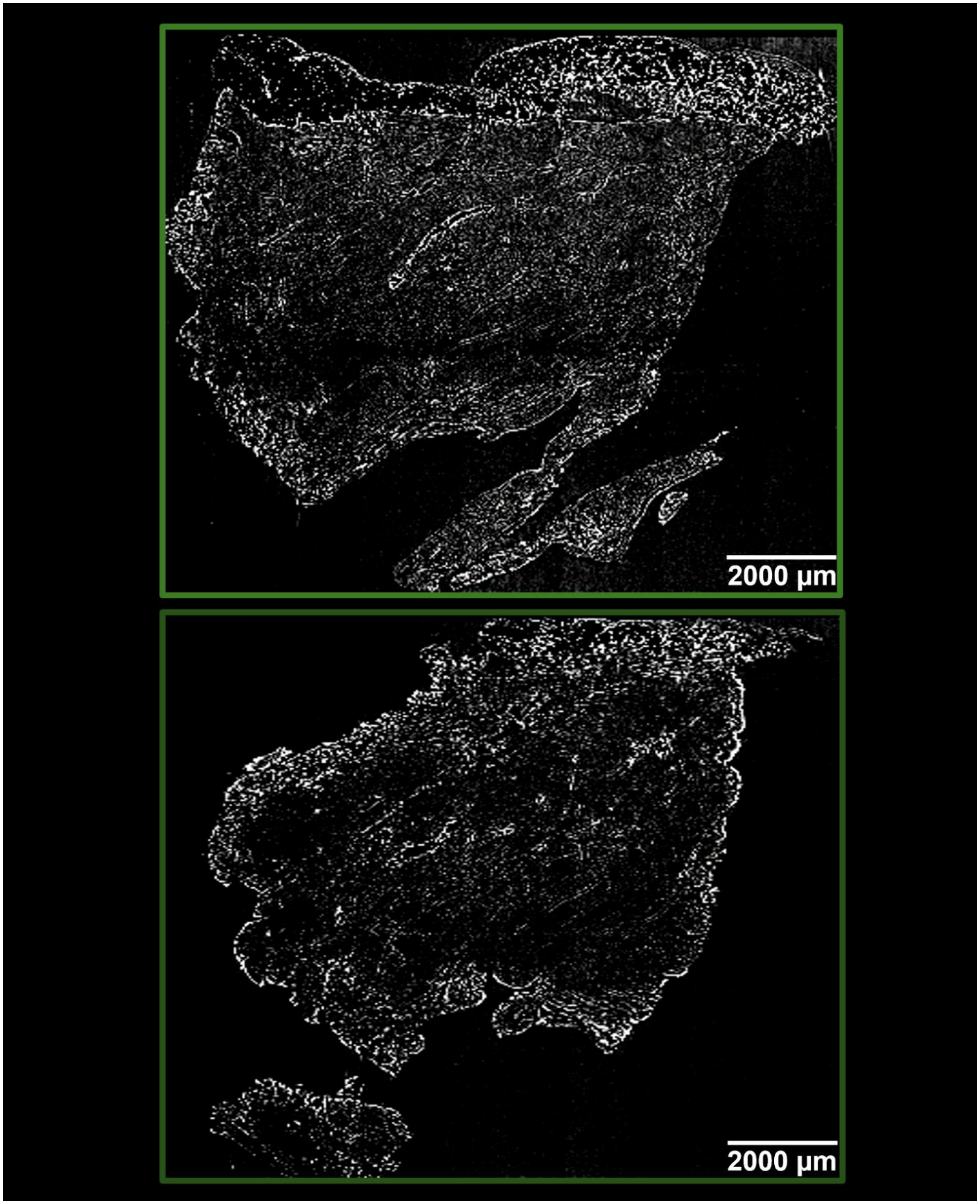

LR=low resolution; ICM=ischaemic cardiomyopathy; LVAD=left ventricular assist device

Supplementary Figure S5. Virtual histopathology of TCM (HTx) sample via X-PCI. Orthogonal views taken from a 3D X-PCI scan of the transmural myocardial tissue sample in a patient undergoing HTx due to toxic cardiomyopathy. The yellow rectangles show selected regions of the myocardium that were scanned with the HR imaging setup enabling analysis of cardiac microstructure. Sparse fibrotic fibrils in the selected HR mid-myocardial region marked in red. The colour-coded horizontal and vertical dotted lines relate to the LR orthogonal cuts of the tissue sample shown in zoomed colour-coded frames in the Supplementary figures S5.2-S5.4 below.

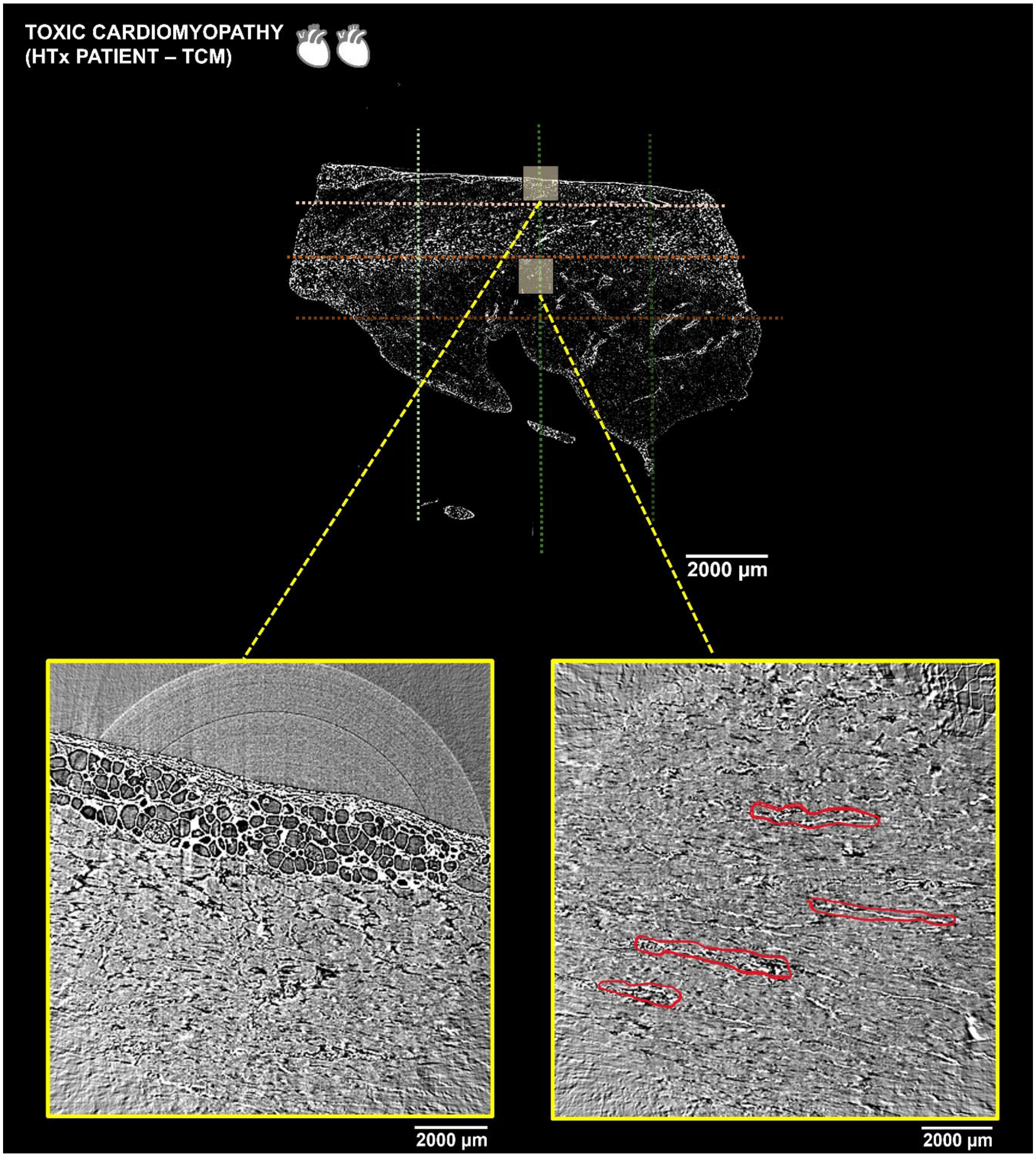

LR=low resolution; TCM=toxic cardiomyopathy; HTx=heart transplantation

Supplementary Figure S5.2. Zoomed beige and orange colour-coded frames of LR orthogonal cuts of TCM (Htx) sample.

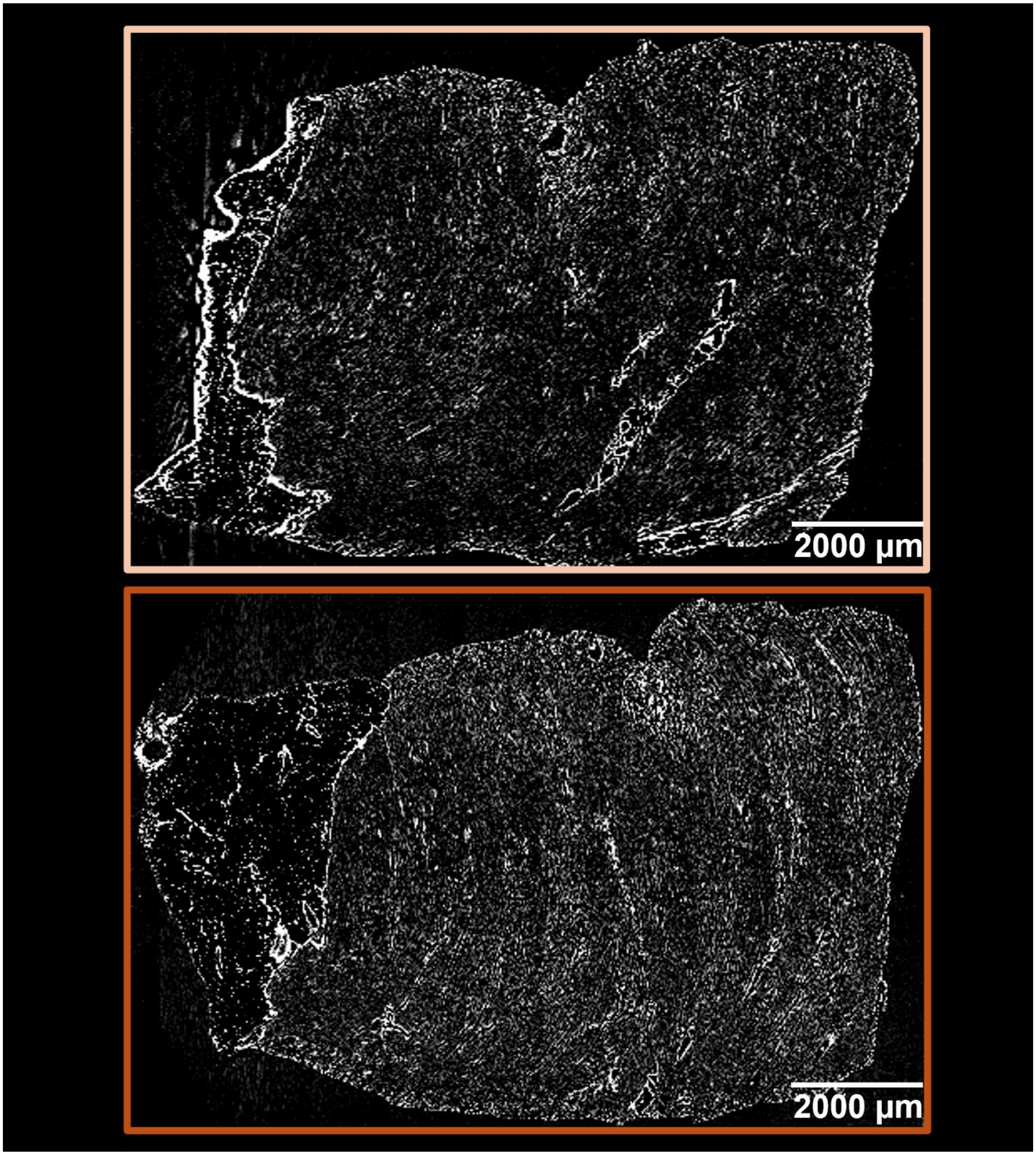

LR=low resolution; TCM=toxic cardiomyopathy; HTx=heart transplantation

Supplementary Figure S5.3. Zoomed brown and sage colour-coded frames of LR orthogonal cuts of TCM (Htx) sample.

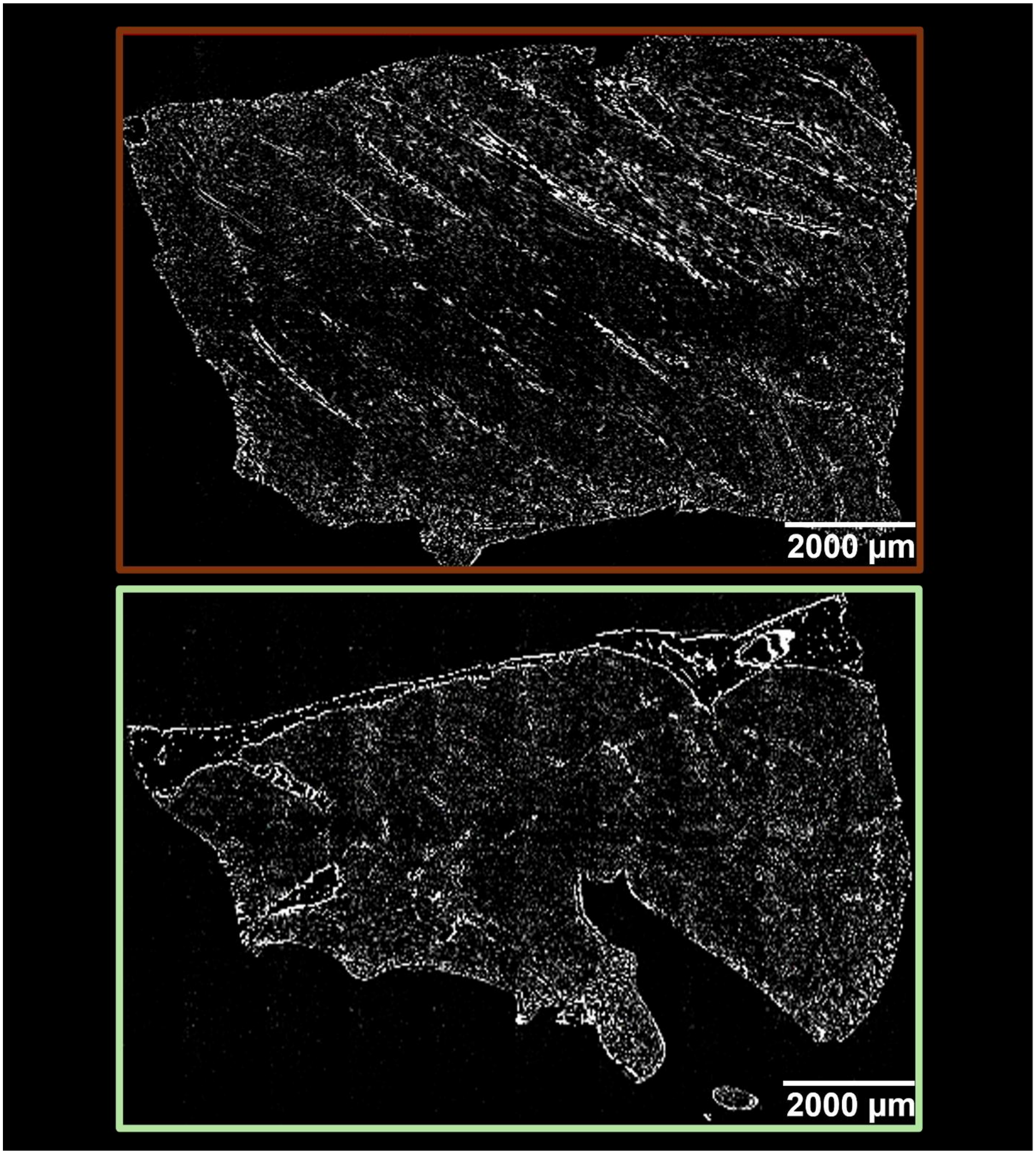

LR=low resolution; TCM=toxic cardiomyopathy; HTx=heart transplantation

Supplementary Figure S5.4. Zoomed green and dark green colour-coded frames of LR orthogonal cuts of TCM (Htx) sample.

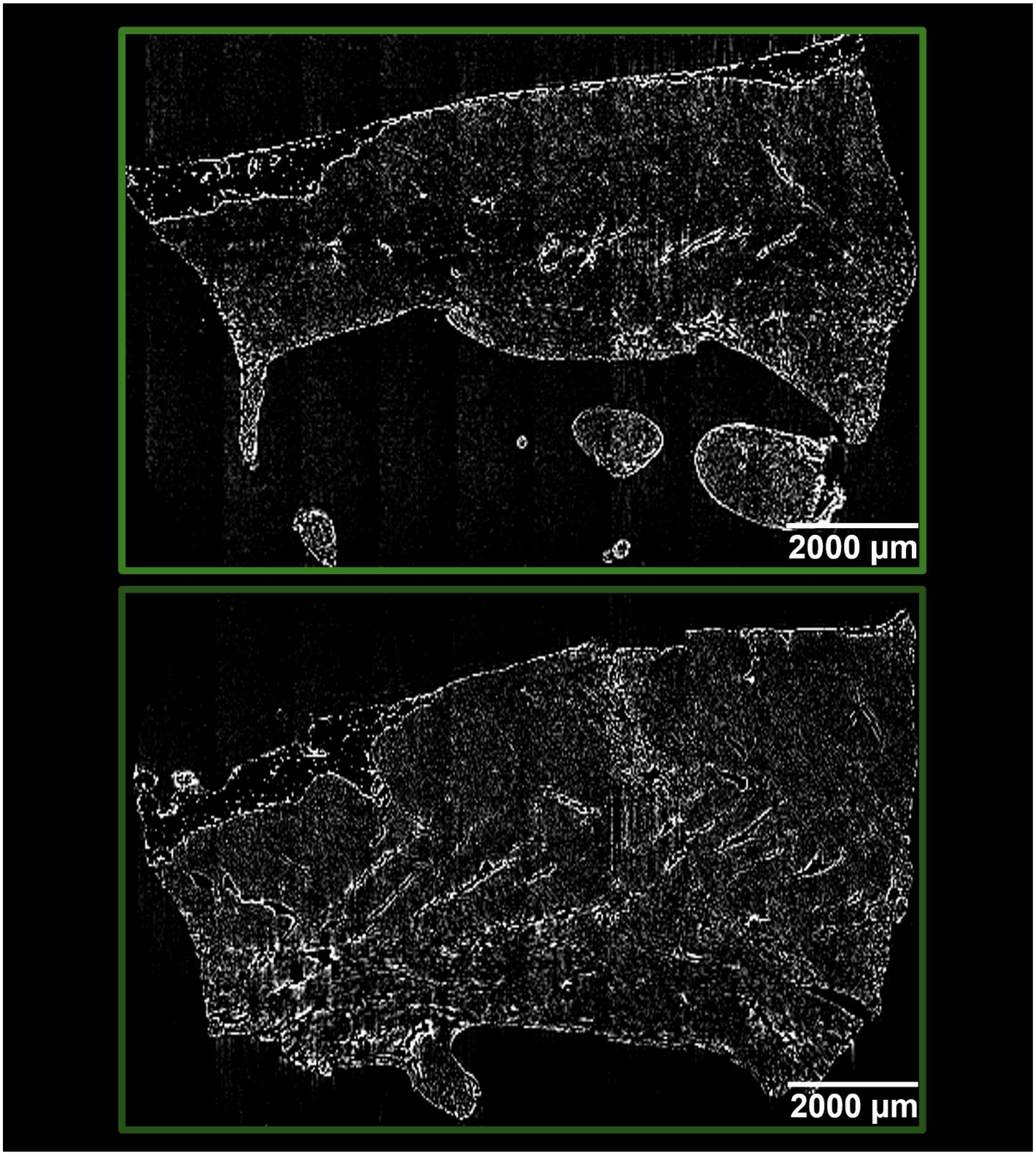

TCM=toxic cardiomyopathy; HTx=heart transplantation; X-PCI=X-ray phase contrast imaging; LR=low resolution; HR=high resolution

Supplementary Figure S6. Schematic of helical angle (HA) and intrusion (also known as transverse) angle in the myocardium used to assess orientation of aggregates of myocytes via structure tensor method[5-7].

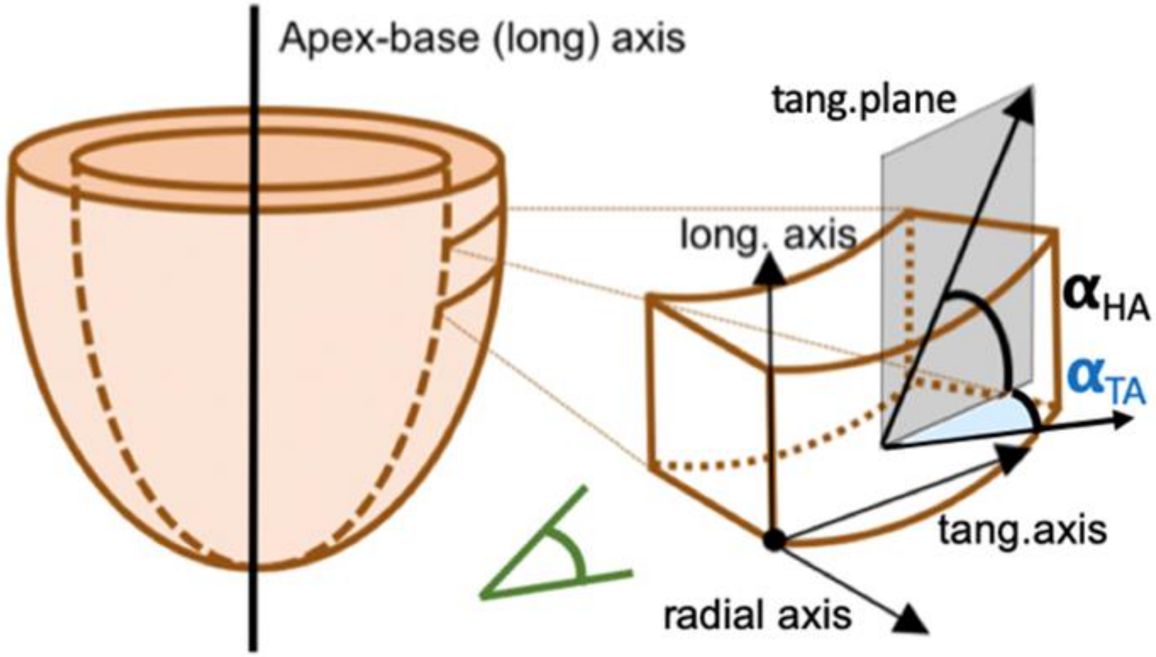

HA=helical angle; TA=transverse angle

Supplementary Figure S7. Pixel classification workflow used for collagen segmentation in software *Ilastik*[9].

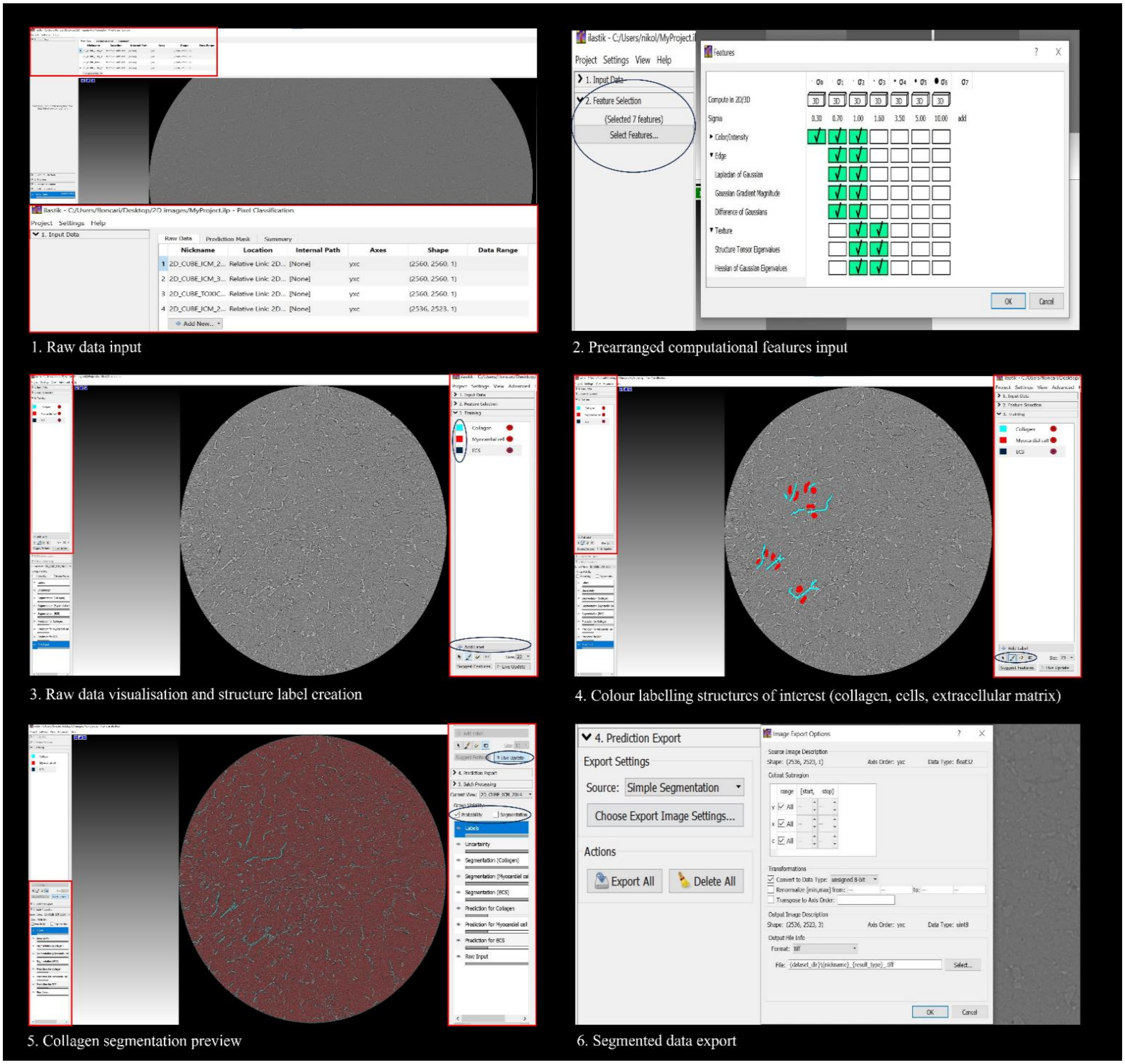

1. Using the left-sided bar select *Input data* for the sample dataset import into the Ilastik software as a TIF sequence.

2. Select *Feature Selection* and input prearranged computational features for the colour/intensity, edge and texture for the image processing and analysis.

3. Select *Training* for the raw dataset visualisation and image setup for segmentation (adjust contrast and brightness on the left-sided bar). Select *Add Label* to create colour labels for the morphological features of interest (collagen, cells, extracellular space).

4. Using a paint brush tool, mark as precise as possible on the part of the image the features of interest as an input for the software algorithm.

5. Select *Prediction* and *Segmentation* command on the left-sided bar to apply collagen segmentation algorithm through the whole dataset. Select *Live Update* for the results preview.

6. When satisfied with the results, select *Prediction Export* and export the segmentation data as a TIF sequence.

Supplementary Tables

Supplementary Table S1. Cardiology panel of 174 tested clinically relevant gene variants, which did not reveal pathogenic mutations in tested patients.

ABCC9	ABCG5	ABCG8	ACTA1	ACTA2
ACTC1	ACTN2	AKAP9	ALMS1	ANK2
ANKRD1	APOA4	APOA5	APOB	APOC2
APOE	BAG3	BRAF	CACNA1C	CACNA2D1
CACNB2	CALM1	CALR3	CASQ2	CAV3
CBL	CBS	CETP	COL3A1	COL5A1
COL5A2	COX15	CREB3L3	CRELD1	CRYAB
CSRP3	CTF1	DES	DMD	DNAJC19
DOLK	DPP6	DSC2	DSG2	DSP
DTNA	EFEMP2	ELN	EMD	EYA4
FBN1	FBN2	FHL1	FHL2	FKRP
FKTN	FXN	GAA	GATAD1	GCKR
GJA5	GLA	GPD1L	GPIHBP1	HADHA
HCN4	HFE	HRAS	HSPB8	ILK
JAG1	JPH2	JUP	KCNA5	KCND3
KCNE1	KCNE2	KCNE3	KCNH2	KCNJ2
KCNJ5	KCNJ8	KCNQ1	KLF10	KRAS
LAMA2	LAMA4	LAMP2	LDB3	LDLR
LDLRAP1	LMF1	LMNA	LPL	LTBP2
MAP2K1	MAP2K2	MIB1	MURC	MYBPC3
MYH11	MYH6	MYH7	MYL2	MYL3
MYLK	MYLK2	MYO6	MYOZ2	MYPN
NEXN	NKX2-5	NODAL	NOTCH1	NPPA
NRAS	PCSK9	PDLIM3	PKP2	PLN
PRDM16	PRKAG2	PRKAR1A	PTPN11	RAF1
RANGRF	RBM20	RYR1	RYR2	SALL4
SCN1B	SCN2B	SCN3B	SCN4B	SCN5A
SCO2	SDHA	SEPN	SGCB	SGCD
SGCG	SHOC2	SLC25A4	SLC2A10	SMAD3
SMAD4	SNTA1	SOS1	SREBF2	TAZ
TBX20	TBX3	TBX5	TCAP	TGFB2
TGFB3	TGFBR1	TGFBR2	TMEM43	TMPO
TNNC1	TNNI3	TNNT2	TPM1	TRDN
TRIM63	TRPM4	TTN	TTR	TXNRD2
VCL	ZBTB17	ZHX3	ZIC3	

References

1. Dejea, H. (2021). *Multiscale and dynamic synchrotron-based tomographic microscopy for cardiovascular applications* (Doctoral thesis, ETH Zurich, Zurich, Switzerland)

2. Marone, F., Stampanoni, M. Regridding reconstruction algorithm for real-time tomographic imaging. J Synchrotron Radiat 2012;**19**:1029–1037

3. Paganin, D., Mayo, S.C., Gureyev, T.E., Miller, P.R., Wilkins, S.W. Simultaneous phase and amplitude extraction from a single defocused image of a homogeneous object. *J Microsc* 2002;**206**:33–40.

4. Garcia-Canadilla, P. et al. Complex Congenital Heart Disease Associated With Disordered Myocardial Architecture in a Midtrimester Human Fetus. *Circ Cardiovasc Imaging* 2018;**11**.

5. Garcia-Canadilla, P. et al. Myoarchitectural disarray of hypertrophic cardiomyopathy begins pre-birth. *J Anat* 2019;**235**:962–976.

6. Garcia-Canadilla, P., Mohun, T.J., Bijnens, B., Cook, A.C. Detailed quantification of cardiac ventricular myocardial architecture in the embryonic and fetal mouse heart by application of structure tensor analysis to high resolution episcopic microscopic data. *Front Cell Dev Biol* 2022;**10**:1000684.

7. Baličević, V. et al. Assessment of Myofiber Orientation in High Resolution Phase-Contrast CT Images. In: Assen H van, Bovendeerd P, Delhaas T, eds. *Functional Imaging and Modeling of the Heart*. Cham: Springer International Publishing; 2015. p111–119.

8. Schindelin, J. et al. Fiji: an open-source platform for biological-image analysis. *Nat Methods* 2012;**9**:676–682.

9. Berg, S. et al. ilastik: interactive machine learning for (bio)image analysis. *Nat Methods* 2019;**16**:1226–1232.

10. Seg3D: Volumetric Image Segmentation and Visualization. https://www.sci.utah.edu/cibc-software/seg3d.html
